# A novel tumor-promoting mechanism of IL6 and the therapeutic efficacy of tocilizumab: Hypoxia-induced IL6 is a potent autophagy initiator in glioblastoma via the p-STAT3-*MIR155-3p*-CREBRF pathway

**DOI:** 10.1080/15548627.2016.1178446

**Published:** 2016-05-10

**Authors:** Hao Xue, Guang Yuan, Xing Guo, Qinglin Liu, Jinsen Zhang, Xiao Gao, Xiaofan Guo, Shugang Xu, Tong Li, Qianqian Shao, Shaofeng Yan, Gang Li

**Affiliations:** a Department of Neurosurgery, Qilu Hospital of Shandong University, Jinan, Shandong Province, China; b Brain Science Research Institute, Shandong University, Jinan, Shandong Province, China; c Department of Neurosurgery, Central Hospital of Zibo City, Zibo, Shandong Province, China; d Department of Neurosurgery, Dezhou People's Hospital, Dezhou, Shandong Province,China; e Institute of Basic Medical Sciences and Key Laboratory of Cardiovascular Proteomics of Shandong Province, Qilu Hospital of Shandong University, Jinan, Shandong Province, China

**Keywords:** ATG5, autophagy, CREB3, CREBRF, glioblastoma, hypoxia, IL6, microRNAs, STAT3, tocilizumab

## Abstract

Hypoxia induces protective autophagy in glioblastoma cells and new therapeutic avenues that target this process may improve the outcome for glioblastoma patients. Recent studies have suggested that the autophagic process is upregulated in glioblastomas in response to extensive hypoxia. Hypoxia also induces the upregulation of a specific set of proteins and microRNAs (miRNAs) in a variety of cell types. IL6 (interleukin 6), an inflammatory autocrine and paracrine cytokine that is overexpressed in glioblastoma, has been reported to be a biomarker for poor prognosis because of its tumor-promoting effects. Here, we describe a novel tumor-promoting mechanism of IL6, whereby hypoxia-induced IL6 acts as a potent initiator of autophagy in glioblastoma via the phosphorylated (p)-STAT3-*MIR155-3p* pathway. IL6 and p-STAT3 levels correlated with the abundance of autophagic cells and HIF1A levels in human glioma tissues and with the grade of human glioma, whereas inhibition of exogenous or endogenous IL6 repressed autophagy in glioblastoma cells in vitro. Knockdown of endogenous *MIR155-3p* inhibited IL6-induced autophagy, and enforced expression of *MIR155-3p* restored the anti-autophagic activity of IL6 inhibitors. We show that the hypoxia-IL6-p-STAT3-*MIR155-3p*-CREBRF-CREB3-ATG5 pathway plays a central role in malignant glioma progression, with blockade of the IL6 receptor by tocilizumab demonstrating a certain level of therapeutic efficacy in a xenograft model in vivo, especially in combination with temozolomide. Moreover, tocilizumab inhibits autophagy by promoting tumor apoptosis. Collectively, our findings provide new insight into the molecular mechanisms underlying hypoxia-induced glioma cell autophagy and point toward a possible efficacious adjuvant therapy for glioblastoma patients.

## Introduction

Autophagy, which means “self-eating,” is a process that involves the formation of double-membrane vesicles containing damaged or old organelles (autophagosomes). After fusion with the lysosome (generating an autolysosome), autophagy provides cells with reclaimed essential elements for survival during starvation, hypoxia, immune response and chemoradiotherapy.^1^ Because cancer cells are overconsumptive, these cells tend to develop ubiquitous autophagy to cope with the relatively infertile tumor microenvironment.^2-4^ Although the autophagic process in normal cells functions as a mechanism that prevents tumorigenesis through the selective cleanup of damaged organelles and specific oncogene proteins, this process is a double-edged sword—autophagy acts as a cytoprotective mechanism in established, advanced tumor cells that have increasing metabolic demands that need to be met.^5,6^ Indeed, accumulating evidence demonstrates that autophagy favors tumor development, and this situation is accordingly a topic of oncological interest.^7^

IL6 (interleukin 6), an inflammatory autocrine and paracrine cytokine overexpressed in glioblastoma multiforme (GBM) and many other malignant tumors,^8-13^ has been reported to be a biomarker for poor prognosis in patients with various types of tumors, such as GBM.^12,13^ This observation may be partially explained by the capacity of IL6 to promote proliferation,^9,11,14^ angiogenesis,^15^ apoptosis,^9^ invasion and migration in different malignancies.^10^ However, the manner in which IL6 contributes to tumor progression by initiating autophagy has yet to be thoroughly investigated.

Because of the rapid growth progression and a relatively inadequate blood supply, hypoxia is a common feature in solid tumors, and tumor hypoxia is an independent prognostic factor associated with poor survival.^16^ Although numerous studies have suggested that hypoxia activates multiple cellular processes in tumors, such as proliferation,^17^ angiogenesis,^18,19^ migration,^20^ invasion,^21^ and autophagy,^22,23^ the mechanisms by which hypoxia induces autophagy remain to be elucidated in detail.

Gliomas are the most malignant type of brain tumor, accounting for more than 70% of all brain tumors.^24^ GBM is the most common type, with an age-adjusted incidence rate that ranges from 0.59 to 3.69 per 100,000 people.^25^ There appears to be general agreement that the properties of GBM largely consist of high mortality and recurrence rates,^26^ uncontrollable invasiveness,^27^ strong angiogenesis,^28^ a widespread hypoxic region,^16,29,30^ increased IL6 expression,^8,13^ and upregulated autophagic processes.^31^ Despite the multitude of mechanisms that have been proposed to explain the hypoxia-induced autophagy of tumor cells, there are no studies evaluating the impact of prominently expressed IL6 on this cellular event.

Members of the STAT (signal transducer and activator of transcription) protein family are important transducers of many cytokines and growth factors in virtually all tumors.^32^ Among the 7 members of the family, STAT3 is the one most commonly constitutively activated in several cancers, including GBM.^33^ It is well accepted that STAT3 serves as a downstream target of IL6, which induces STAT3 activation through tyrosine 705 phosphorylation and cytoplasmic-to-nuclear shuttling, recognition of STAT3-specific DNA motifs, and transcriptional activation of target genes that are implicated throughout tumor development and involved in proliferation, survival, invasion and angiogenesis.^34,35^ Recent studies have demonstrated that STAT3 also regulates the expression of several microRNAs (miRNAs). miRNAs comprise a class of noncoding endogenous RNAs that regulate the expression of genes that participate in almost every biological process by binding to the 3′-untranslated region (3′-UTR) of target protein-coding mRNA molecules.^36^ Moreover, several miRNAs have been well characterized as being involved in autophagy regulation.^37^ Indeed, there is increasing evidence that miRNAs might be the link between hypoxia-induced IL6 levels and the enhancement of tumor autophagy.

CREBRF/LRF (CREB3 regulatory factor) is a novel cellular protein and a negative regulator of CREB3/Luman/LZIP (cAMP responsive element binding protein 3) that can recruit nuclear CREB3 to discrete foci in the nucleus, promote CREB3 protein degradation and repress CREB3-mediated activation of unfolded protein response element-containing promoters.^38^ CREB3 is the primary member of the CREB3 family. All CREB3 family members appear to play a role in the unfolded protein response,^39^ during which endoplasmic reticulum (ER)-resident molecular chaperones and foldases are induced, attenuating translation to reduce the load on the ER.^40^ Unfolded proteins can also be targeted for proteasomal degradation via ubiquitination.^41^ CREB family proteins, including CREB1, CREB2 and CREB3, bind to the cAMP-responsive element recognition sequence of cAMP-sensitive genes to regulate transcription. Although it has been reported that CREB1 can upregulate autophagy genes,^42,43^ and that CREBRF is involved in inducing cell apoptosis through the ER stress pathway,^44^ there is no evidence to date that the CREBRF-CREB3 pathway is involved in regulating autophagy in tumor cells.

In the present study, we report a novel tumor-promoting mechanism of IL6 in which hypoxia-induced IL6 promotes tumor cell survival by upregulating autophagy in GBM through the p-STAT3-*MIR155-3p* pathway. We first investigated the considerable initiating effect of IL6 during the hypoxia process, and we found that hypoxic pretreatment of tumor cells induced significant IL6 expression and autophagy activation. More importantly, the application of exogenous IL6 increased autophagic activity, whereas knocking down endogenous IL6 or treatment with IL6 antibodies alleviated hypoxia-induced autophagy. To understand the mechanisms of the autophagy induced by IL6, we screened the entire complement of genomic miRNAs using gene chips (Human miRCURY™ LNA expression array). Analysis of the data revealed dramatic changes in multiple molecules under hypoxia, especially those related to IL6 and autophagy. Based on these results, we selected the molecules downstream of IL6 implicated in the autophagic process for further examination. Finally, we provide evidence that the p-STAT3-*MIR155-3p* pathway plays a central role in the impact of IL6. Our results suggest potential uses for anti-IL6 therapeutic strategies in adjuvant therapy for glioma patients. In a broader sense, the data also support the application of a monoclonal antibody to block the hypoxia-IL6-p-STAT3-*MIR155-3p*-CREBRF-CREB3-ATG5 pathway at the source. Several attempts have been made to therapeutically regulate the autophagic-promoting activity of IL6, and we were encouraged by the fact that tocilizumab, a novel monoclonal antibody injectable for rheumatoid arthritis that targets IL6, could achieve this therapeutic effect through specific blockade of the IL6 receptor. We conducted a series of successful tocilizumab therapeutic experiments using glioma cell xenograft mouse models, especially in combination with temozolomide; however, further clinical investigations are needed.

## Results

### IL6 levels correlated with the density of autophagic cells, HIF1A levels in human glioma tissues and the grade of human glioma

**Figure 1.**
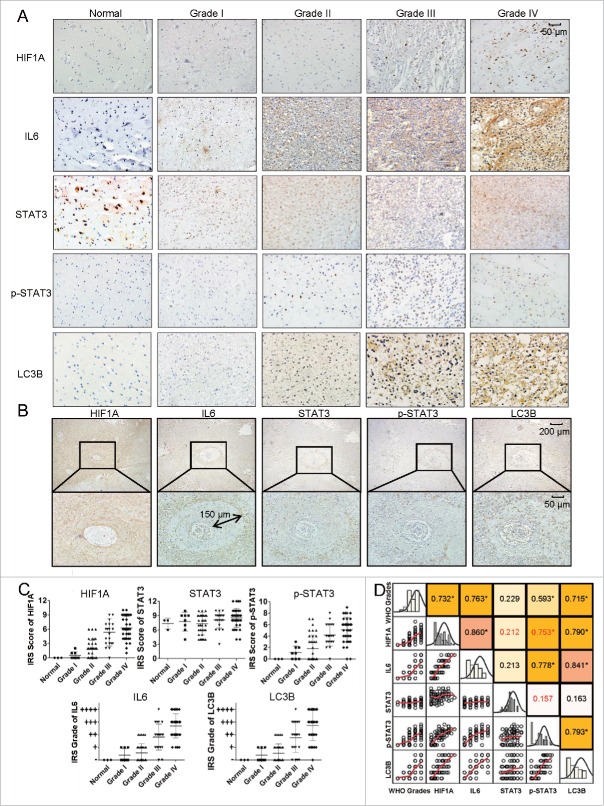
Levels of colocalized IL6, HIF1A, LC3B and p-STAT3 positively correlate with the WHO grade in human glioma tissues. (A) The expression of IL6, HIF1A, p-STAT3, and LC3B positively correlated with the WHO grade of gliomas. Ninety human gliomas and 3 normal brain tissue samples of different WHO grades were processed by immunohistochemistry. STAT3 levels were basically stable. (B) Immunohistochemical staining of continuous paraffin sections revealed the colocalization of IL6, p-STAT3, HIF1A, and LC3B proteins in the hypoxic area around the tumor vessels of a sample of high-grade glioma tissues (WHO III) (enlarged view of the lower right green box in Fig. S1). (C) Quantification of IHC staining of glioma tissues with different WHO grades. IRS, immunoreactive score. (D) Pearson's or Spearman's rho rank correlation tests of IL6, p-STAT3, HIF1A, LC3B and grades.

Extensive hypoxic areas have been found in glioma tissues;^45^ however, the relationship between IL6 and the level of autophagy in the hypoxic areas of tumors remains unclear. In fact, a causal link between IL6 and LC3B levels and hypoxia levels in gliomas has not been reported, and it is also unknown whether IL6 is involved in the upregulation of autophagy in such hypoxic areas. To examine whether the expression of IL6 correlates with autophagy and the level of hypoxia in human glioma, we performed immunohistochemical staining to detect the expression of IL6, STAT3 and p-STAT3 (the primary downstream signaling molecules of IL6), LC3B (a marker of autophagy) and HIF1A in 101 human glioma specimens with different grades and 3 normal brain tissues (Table 1). As shown in Fig. 1A, the expression of IL6, p-STAT3, HIF1A, and LC3B positively correlated with the WHO grade of glioma (Fig. 1C), and Pearson's or Spearman's rho rank correlation tests showed that IL6, p-STAT3, HIF1A, LC3B and grade were positively correlated. Most correlation indexes were approximately 0.7 to 0.8, indicating a strong positive relationship (Fig. 1D). In addition, the expression of the IL6-p-STAT3 pathway strongly correlated with LC3B and HIF1A levels; consistent with this finding, the abundance of autophagic cells was also associated with HIF1A levels (Fig. 1A). Furthermore, immunohistochemical staining of continuous paraffin sections revealed the colocalization of the IL6, p-STAT3, HIF1A, and LC3B proteins in high-grade glioma tissues (Fig. S1), particularly in the hypoxic area around tumor vessels (Fig. 1B, the enlarged view of the lower right green box in Fig. S1). The maximum diffusion distance of the oxygen supply around the tumor vessel in solid tumors was approximately 100 to 150 μm,^46^ which agreed with our staining results and illustrated the hypoxic regions around tumor vessels (Fig. 1B). Taken together, these results demonstrate that IL6 expression correlates with the density of autophagic cells and HIF1A levels in high-grade (WHO III and IV) gliomas.

To further investigate tumor-associated cells in this hypoxic glioma microenvironment and to clarify the cellular sources of IL6, we collected 5 freshly removed, surgical WHO grade IV glioma tissue samples for fast-frozen sectioning, and staining revealed most of the tumor-associated cells in the microenvironment, including T cells (CD3), macrophages (CD68, CD11B), microglia (CD68), M1 tumor-associated macrophages (CD11B, CD16), M2 tumor-associated macrophages (CD206), tumor-associated dendritic cells (CD11C), monocytes and some NK cells (CD16) (Fig. S2A). These tumor-associated cells accounted for approximately 30% of all cells in the glioma tissues, as some of the markers overlapped. Importantly, the IL6-positive cells accounted for over 90% of all cells. As IL6 can be derived from many sources, including glioma cells and macrophages, we double stained these markers and found that the IL6 was primarily secreted from tumor cells (Fig. S2C), with only 25% of the total amount of IL6 being secreted by nontumor immune cells (Fig. S2D). Thus, glioma cells are clearly stimulated by strong IL6 signals in a complex hypoxic microenvironment.

### Hypoxia induces autophagy activation and IL6 upregulation in glioblastoma cells

**Figure 2.**
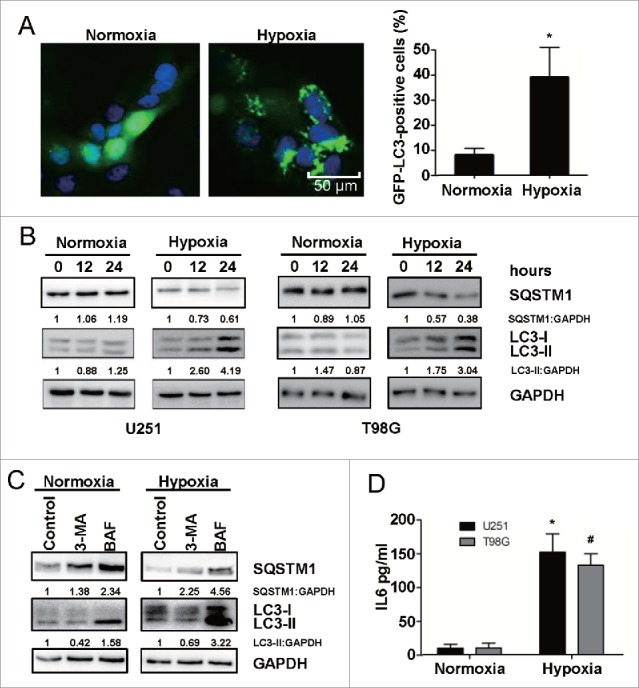
Hypoxia induces autophagy activation and upregulates IL6 in human glioma cells. (A) Hypoxia promotes GFP-LC3B translocation. pSELECT-GFP-LC3B transfection revealed LC3B puncta in U251 cells treated with hypoxia (1% O_2_, 5% CO_2_, and 94% N_2_ at 37°C) for 24 h. Cells were fixed and stained with DAPI for nuclear visualization. Representative images are shown. Scale bar: 50 μm. Quantitative analysis of GFP-LC3B puncta is shown in the right panel. At least 100 high-power fields were examined in each experimental group. The data shown are the mean ± s.d. of 4 independent experiments. *, P < 0.0001; 2-tailed t test. (B) Hypoxia induced LC3B conversion and SQSTM1 degradation in U251 and T98G cells. LC3B and SQSTM1 levels were examined by western blot analysis in GBM cells after hypoxia treatment (1% O_2_, 5% CO_2_, and 94% N_2_ at 37°C) for 12 and 24 h. GAPDH served as the loading control. (C) Western blot analysis showing that 3-methyladenine (3-MA) inhibited autophagy in U251 cells and treatment of normoxic and hypoxic cells with bafilomycin (BAF) blocked autophagic flux. (D) Hypoxia increased the secretion of IL6 in U251 and T98G cell culture supernatants, as revealed by ELISA. The data shown are the mean ± s.d. of 3 independent experiments. * and ^#^, P < 0.001; 2-tailed t test.

It is generally accepted that hypoxia activates autophagy as an evolutionarily conserved cellular catabolic process.^23^ To verify this, we performed a GFP-LC3B puncta-formation assay and an LC3B conversion assay. Using GBM cells (U251) stably expressing a GFP-LC3B fusion protein, GFP-LC3B localization was examined by fluorescence microscopy. GFP-LC3B puncta appearing in the cytoplasm reflect the recruitment of the LC3B protein to phagophores, the precursor to autophagosomes. As shown in Fig. 2A, there was a significant increase in GFP-LC3B puncta in hypoxic cells, a result that was confirmed by the quantification of GFP-LC3B foci per cell. Moreover, we detected the conversion of LC3B-I to LC3B-II and the degradation of SQSTM1/p62 (autophagy markers) by western blotting. Consistent with the GFP-LC3B puncta-formation assay, hypoxia led to a significant time-dependent upregulation of LC3B-II and downregulation of SQSTM1 (Fig. 2B). Thus, both assays suggested that hypoxia induces autophagosome accumulation. In addition, treatment of normoxic and hypoxic cells with 3-methyladenine (3-MA) inhibited the autophagy (Fig. 2C),^47^ as previously observed, whereas treatment of normoxic and hypoxic cells with bafilomycin A_1_ (BAF) blocked autophagic flux and resulted in an increase in hypoxia-induced autophagy (Fig. 2C).^48^ To investigate the function of IL6 in hypoxia-induced autophagy, we used ELISA to examine the secretion of IL6 in a GBM cell culture supernatant (U251) under hypoxic stress. As shown in Fig. 2D, the secretion level of IL6 was low under normal culture conditions (21% oxygen), but hypoxia (1% oxygen) treatment induced a significant increase in IL6 secretion.

### Exogenous IL6 triggers autophagy and inhibition of endogenous IL6 represses autophagy in glioblastoma cells

**Figure 3.**
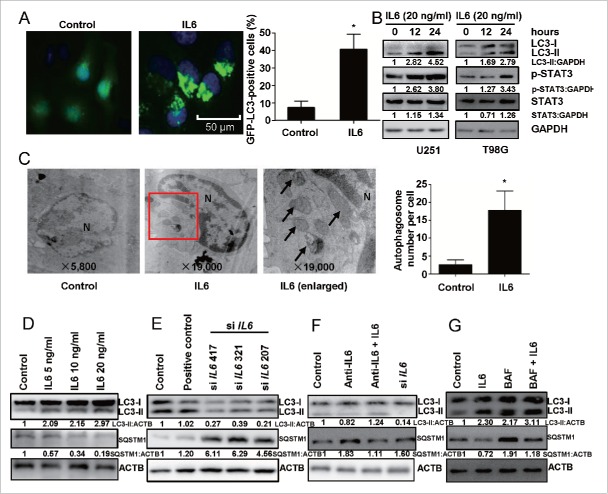
IL6 induces autophagy activation, and inhibition of IL6 represses autophagy in human glioma cells. (A) Exogenous IL6 promotes GFP-LC3B translocation. pSELECT-GFP-LC3B-transfected U251 cells treated with IL6 (20 ng/ml) for 24 h. Scale bar: 50 μm. Quantitative analysis of GFP-LC3B puncta is shown in the right panel. The data shown are the mean ± s.d. of 4 independent experiments. *, P < 0.0001; 2-tailed t test. (B) Exogenous IL6 induced LC3B conversion and STAT3 activation in U251 and T98G cells. LC3B, STAT3 and p-STAT3 levels were examined by western blot analysis in GBM cells after treatment with IL6 (20 ng/ml) for 0, 12, 24 h. GAPDH served as the loading control. (C) Images from transmission electron microscopy showing characteristic autophagosomes (arrows) in U251 cells after treatment with IL6 (20 ng/ml) for 24 h. N, nucleus. At least 50 cells were examined in each experimental group. The data shown are the mean ± s.d. of 3 independent experiments. *, P < 0.0001; 2-tailed t test. (D) Exogenous IL6 induces LC3B conversion and SQSTM1 degradation in GBM cells in a dose-dependent manner after a 24-h treatment. Inhibition of endogenous IL6 (E) and exogenous IL6 (F) represses LC3B conversion and SQSTM1 degradation in GBM cells as measured by western blot analysis. (G) Autophagic flux inhibition of IL6-treated U251 cells with bafilomycin A_1_ (BAF). ACTB served as the loading control.

An increasing number of studies have shown that cancer cells, including GBM cells, undergo autophagy in response to hypoxia,^45^ yet it remains unknown whether hypoxia-induced IL6 is a central mediator of hypoxia-induced autophagy. Thus, we examined whether IL6 could independently induce autophagy in GBM cells by utilizing GFP-LC3B transient transfection, the LC3B conversion assay and transmission electron microscopy to visualize the aggregation of expressed LC3B in U251 cells. We determined the induction of autophagy through the localization of the autophagosome-specific protein LC3B using GFP-LC3B transient transfection. As shown in Fig. 3A, we observed abundant autophagosomes in IL6-treated cells. Moreover, a western blot analysis was performed to examine whether IL6 treatment induced the processing of LC3B-I to LC3B-II, and the level of the latter increased with the duration of IL6 treatment (Fig. 3B). This effect was observed to occur in a dose-dependent manner in IL6 cells treated for 24 h. In contrast, the opposite trend was observed for SQSTM1 (Fig. 3D), the downregulation of which indicates the activation of autophagy.^49^ Transmission electron microscopy also revealed abundant characteristic autophagosomes in IL6-treated U251 cells (Fig. 3C). To further support the fact that both endogenous IL6 and exogenous IL6 trigger autophagy, we examined LC3B-II and SQSTM1 protein levels after attenuating IL6 activity by small interfering RNA (siRNA) and the use of a recombinant human antibody. The data showed that a blockade of both endogenous and exogenous IL6 repressed autophagy in GBM cells (Fig. 3E, F). Furthermore, to test whether the IL6-induced increase in LC3B-II was due to autophagy induction or the inhibition of autolysosome function, BAF was used to inhibit autophagic flux. As shown in Fig. 3G, although LC3B-II levels increased in BAF-treated cells due to inhibition of LC3B-II lysosomal degradation, the LC3B-II level was even higher in cells additionally treated with IL6. We also found that the expression of SQSTM1 was decreased in IL6-treated cells in the absence of BAF, suggesting that autophagy was activated and that SQSTM1 was degraded via autophagy. The elevated level of SQSTM1 in cells treated with BAF and IL6 indicated that autophagy was blocked by BAF and that SQSTM1 accumulated in GBM cells (Fig. 3G).

### Activation of the IL6-p-STAT3 pathway is involved in hypoxia-induced autophagy in glioblastoma cells

**Figure 4.**
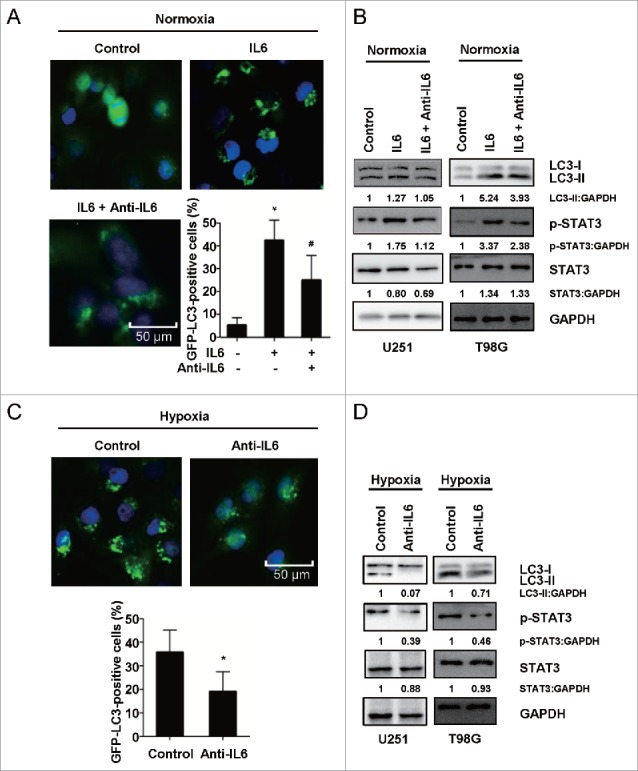
Activation of the IL6-p-STAT3 pathway is involved in hypoxia-induced autophagy in glioblastoma cells. (A) An antibody against exogenous IL6 inhibited GFP-LC3B translocation. pSELECT-GFP-LC3B-transfected U251 cells treated with IL6 (20 ng/ml) and an IL6 antibody (1 μg/ml) for 24 h. Scale bar: 50 μm. Quantitative analysis of GFP-LC3B puncta is shown in the right panel. The data shown are the mean ± s.d. of 4 independent experiments. * and ^#^, P<0.001; one-way ANOVA. (B) An antibody against exogenous IL6 inhibited LC3B conversion and STAT3 activation in U251 and T98G cells. LC3B, STAT3 and p-STAT3 levels were examined by western blot analysis in GBM cells after treatment with IL6 (20 ng/ml) and an IL6 antibody (1 μg/ml) for 24 h. GAPDH served as the loading control. (C) An antibody against exogenous IL6 inhibited GFP-LC3B translocation in hypoxic U251 cells. pSELECT-GFP-LC3B-transfected U251 cells treated with IL6 antibody (1 μg/ml) for 24 h under hypoxic conditions. Scale bar: 50 μm. The quantitative analysis of GFP-LC3B puncta is shown in the right panel. The data shown are the mean ± s.d. of 4 independent experiments. *, P < 0.0001; 2-tailed t test. (D) An antibody against exogenous IL6 inhibited LC3B conversion and STAT3 activation in hypoxic U251 and T98G cells. LC3B, STAT3 and p-STAT3 levels were examined by western blot analysis after treatment of hypoxic GBM cells with an IL6 antibody (1 μg/ml) for 24 h. GAPDH served as the loading control.

As discussed above, IL6 was shown to exert a facilitative influence on the activation of autophagy in GBM cells, and hypoxia could elevate IL6 secretion, suggesting that IL6 may be a crucial mediator of hypoxia-induced autophagy. Using U251 cells transiently transfected with GFP-LC3B, we found that under normoxic conditions, exogenous IL6 induced intense autophagy, similar to hypoxia alone (Fig. 4A, C). Moreover, we observed that a recombinant human IL6 antibody dramatically impaired IL6-induced autophagy and even hypoxia-induced autophagy (Fig. 4). The results of western blot analysis suggested that the IL6 antibody inhibited hypoxia-induced autophagy by blocking the IL6-p-STAT3 pathway (Fig. 4B and D). Additionally, *IL6* siRNA against endogenous *IL6* also blocked activation of the IL6-p-STAT3 pathway and hypoxia-induced autophagy in glioblastoma cells (Fig. S3).

### *MiR155-3p* is involved in IL6-induced autophagy in hypoxic glioblastoma cells

**Figure 5.**
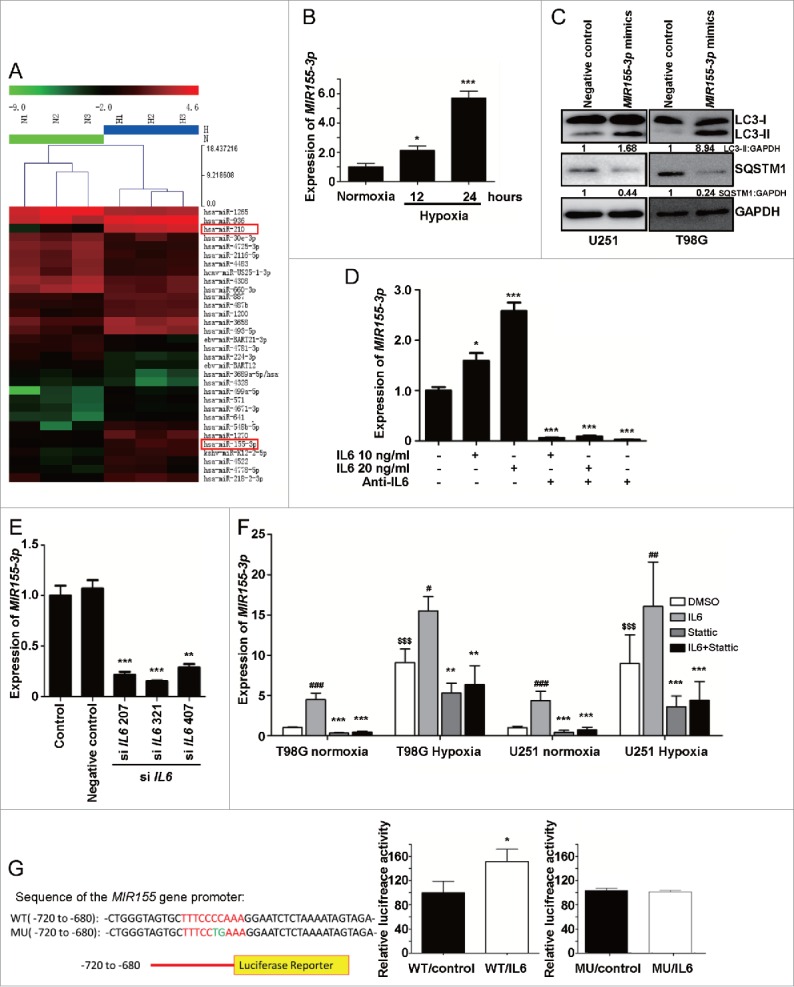
*MIR155-3p* is upregulated by hypoxia, and IL6 can induce autophagy in glioblastoma cells. (A) The miRCURY™ RNA expression array revealed 84 significantly differentially expressed miRNAs (partial data shown in Fig. 5A) between normoxic and hypoxic U251 cells. The hypoxic miRNA marker *MIR210* and the target miRNA *MIR155-3p* are indicated. (B) The expression levels of *MIR155-3p* in hypoxic U251 cells (hypoxia treatment for 0, 12, and 24 h) were assessed by quantitative real-time PCR. The data shown are the mean ± s.d. of 5 independent experiments. *, P < 0.05; ***, P < 0.0001; one-way ANOVA. (C) *MIR155-3p* overexpression induced LC3B conversion and SQSTM1 degradation in U251 and T98G cells at 48 h after *MIR155-3p* mimic transfection, as shown by western blot analysis. GAPDH served as the loading control. (D) Exogenous IL6 upregulated and an antibody against exogenous IL6 inhibited the expression levels of *MIR155-3p*, as determined by quantitative real-time PCR. The data shown are the mean ± s.d. of 5 independent experiments. *, P < 0.01; ***, P < 0.0001; one-way ANOVA. (E) Knockdown of endogenous IL6 inhibited the expression of *MIR155-3p*, as determined by quantitative real-time PCR. (F) The specific p-STAT3 inhibitor Stattic inhibited the function of STAT3. (G) A “5′-TTTCCCCAAA-3′” p-STAT3-binding element is present at −731 in the *MIR155-3p* promoter. Mutation of the p-STAT3-binding element eliminated the promoting effect of the IL6-p-STAT3 pathway on *MIR155-3p* expression compared with the wild-type element. The data shown are the mean ± s.d. of 5 independent experiments. * and ^#^, P < 0.01; ** and ^##^, P < 0.001; ***, ^###^ and ^$$$^, P < 0.0001; * by Student's t-test for Stattic groups versus IL6 groups, ^#^ by the Student *t* test for IL6 groups vs. DMSO control groups, and ^$^ by Student *t* test for hypoxia groups versus normoxia groups.

Because several miRNAs have been well characterized as modulators of autophagy and hypoxia is an independent autophagy-promoting factor, we used a normoxic and hypoxic U251 cell miRNA microarray to identify hypoxia-induced miRNAs. These data revealed 84 significantly differentially expressed miRNAs, including *MIR210*, a novel miRNA marker of hypoxia, and *MIR155-3p*, the homolog of which (*MIR155-5p*) is a potent inducer of autophagy via MTOR pathway targeting (Fig. 5A and C).^37^ We further verified the levels of *MIR155-3p* in hypoxic U251 cells by quantitative real-time PCR, and the validated expression results were consistent with the microarray results. *MIR155-3p* expression was time dependent in hypoxia-treated U251 cells (Fig. 5B) and dose dependent in IL6-treated cells (Fig. 5D). To further investigate whether *MIR155-3p* and IL6 are linked, we utilized siRNA and a recombinant human antibody that has been previously demonstrated to interfere with IL6. As shown in Figure 5D and E , suppression of IL6 significantly reduced *MIR155-3p* expression.

To further examine the relationship between p-STAT3 and *MIR155-3p*, we utilized a specific p-STAT3 inhibitor, Stattic, to inhibit the function of STAT3. We found that Stattic blocked the IL6- and hypoxia-induced upregulation of *MIR155-3p* in U251 and T98G glioma cells (Fig. 5F). We then searched for p-STAT3-binding elements in the sequence of the *MIR155-3p* promoter and identified a “5′-TTTCCCCAAA-3′” element at −731. To assess the direct promoting effect of p-STAT3 on the *MIR155-3p* promoter, p-STAT3-binding element mutation and luciferase reporter assays were performed using U251 glioma cells. Mutation of the p-STAT3-binding element eliminated the promoting effect of the IL6-p-STAT3 pathway on *MIR155-3p* expression compared with the wild-type element (Fig. 5G). These results suggested that *MIR155-3p* may be involved in regulating the autophagic process downstream of the IL6-p-STAT3 pathway.

### *MIR155-3p* knockdown antagonizes hypoxia-induced autophagy and the pro-autophagic effects of IL6 on human glioma cells

**Figure 6.**
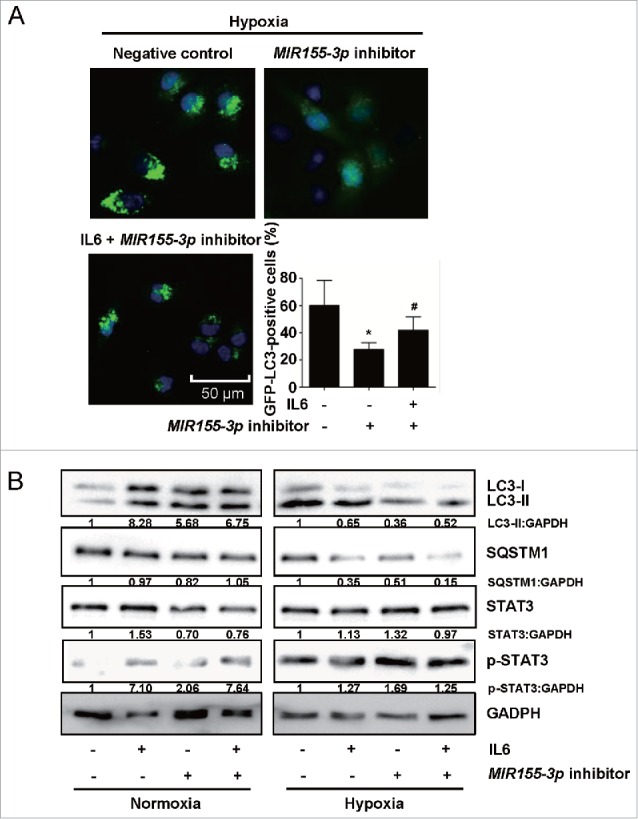
*MIR155-3p* knockdown antagonizes hypoxia-induced autophagy in human glioma cells. (A) The *MIR155-3p* inhibitor suppressed GFP-LC3B translocation. pSELECT-GFP-LC3B and *MIR155-3p* inhibitor-cotransfected U251 cells were treated with IL6 (20 ng/ml) for 24 h. Scale bar: 50 μm. The quantitative analysis of GFP-LC3B puncta is shown in the right panel. The data shown are the mean ± s.d. of 4 independent experiments. * and ^#^, P < 0.001; one-way ANOVA. (B) *MIR155-3p* inhibition suppresses LC3B conversion and STAT3 activation in U251 and T98G cells. LC3B, SQSTM1, STAT3 and p-STAT3 levels were examined by western blot analysis in GBM cells after treatment with IL6 (20 ng/ml) for 24 h. GAPDH served as the loading control.

To investigate whether the downregulation of *MIR155-3p* could affect hypoxia-mediated glioma cell autophagy, we used a *MIR155-3p* inhibitor. miRNA inhibitors are synthetic, 2′-O-methyl-modified, single-stranded molecules that interfere with miRNA function by sequestering these molecules via irreversible binding.^50,51^ The GFP-LC3B puncta-formation assay showed that the *MIR155-3p* inhibitor significantly suppressed U251 cell autophagy under hypoxic conditions, an effect that was partially rescued by exogenous IL6 (Fig. 6A). The results of western blot analysis clearly indicated that *MIR155-3p* knockdown blocked the IL6-induced increase in LC3B-II as well as SQSTM1 degradation under hypoxic conditions (Fig. 6B).

### *MIR155-3p* enhances hypoxia-induced autophagy and rescues the anti-autophagic effects of IL6 inhibition on human glioma cells

**Figure 7.**
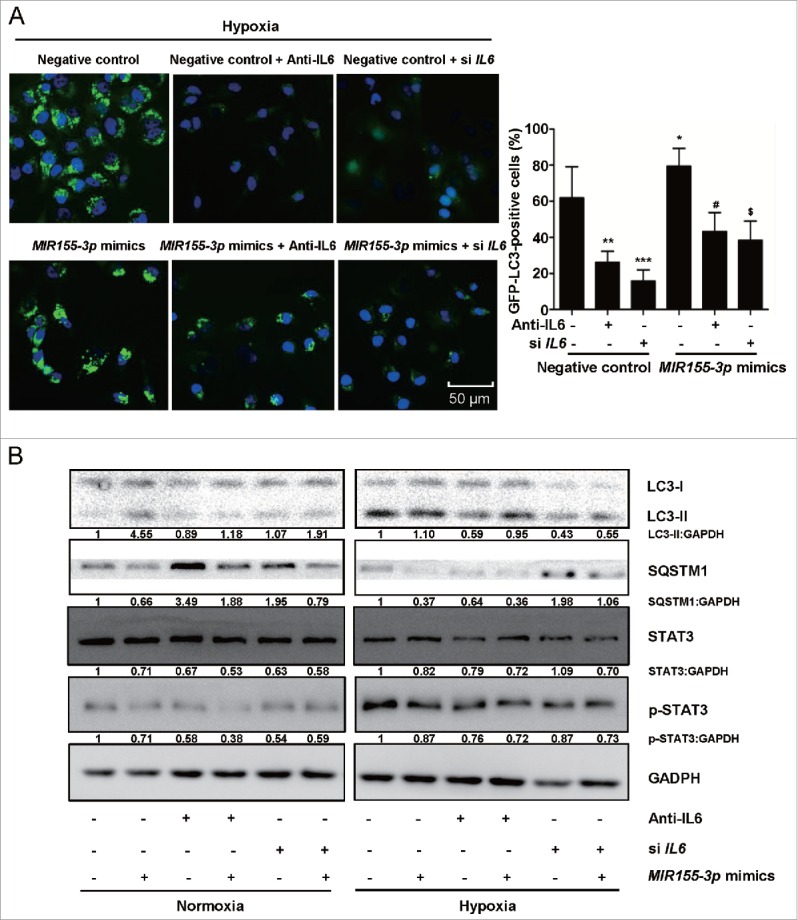
*MIR155-3p* overexpression enhances hypoxia-induced autophagy and resists the effects of IL6 inhibition on human glioma cells. (A) *MIR155-3p* mimic promoted GFP-LC3B translocation. pSELECT-GFP-LC3B and *MIR155-3p* mimic-co-transfected U251 cells treated with the IL6 antibody (1 μg/ml) and siRNAs (Si IL6 321) for 24 h. Scale bar: 50 μm. Quantitative analysis of GFP-LC3B puncta is shown in the right panel. The data shown are the mean ± s.d. of 4 independent experiments. *, ^#^ and ^$^, P < 0.01; **, P < 0.001; ***, P < 0.0001; one-way ANOVA. (B) The *MIR155-3p* mimic induced LC3B conversion and STAT3 activation in U251 and T98G cells. LC3B, SQSTM1, STAT3 and p-STAT3 levels were examined by western blot analysis. GAPDH served as the loading control.

To further understand the pro-autophagic role of *MIR155-3p*, we investigated the effects of its overexpression using miRNA mimics, which consist of synthetic, double-stranded, modified RNA molecules that imitate the functions of endogenous miRNAs.^52^ The GFP-LC3B puncta-formation assay showed that the *MIR155-3p* mimics further enhanced glioma cell autophagy under hypoxic conditions, partially rescuing the anti-autophagic effects of *IL6* siRNA and the recombinant human IL6 antibody (Fig. 7A). Western blot analysis of the LC3B conversion assay showed that overexpression of *MIR155-3p* had pro-autophagic effects similar to exogenous IL6 and rescued the anti-autophagic effects of the IL6 antibody and siRNA by upregulating LC3B-II levels and downregulating SQSTM1 levels, respectively, particularly under hypoxic conditions (Fig. 7B).

### *MIR155-3p* enhances hypoxia-induced autophagy by directly targeting the CREBRF-CREB3-ATG5 pathway in human glioma cells

**Figure 8.**
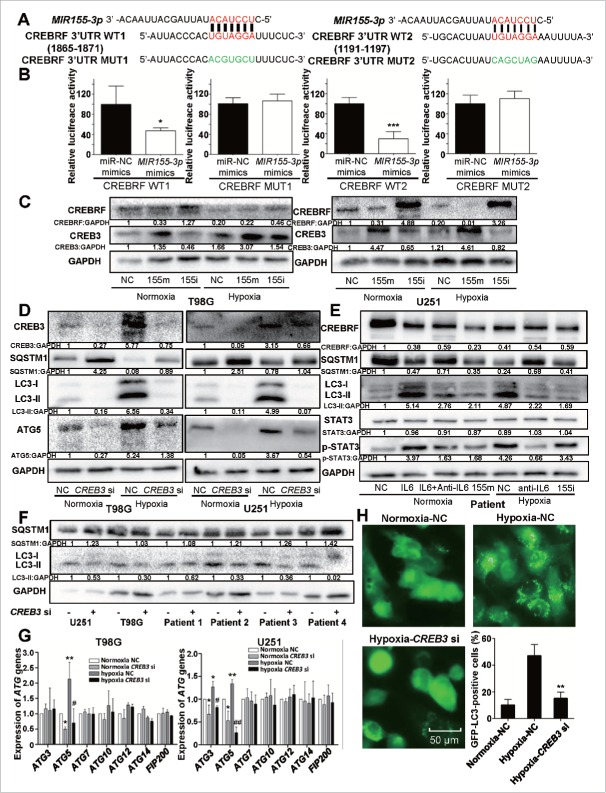
*MIR155-3p* enhances hypoxia-induced autophagy by directly targeting the CREBRF-CREB3-ATG5 pathway in human glioma cells. (A) The best potential *MIR155-3p* target gene, CREBRF, in miRDB, and the 3′-UTR seed mutation. (B) The 3′-UTR seed mutation and 3′-UTR luciferase assays in U251 glioma cells. The luciferase activity in *MIR155-3p*-transfected cells decreased to approximately half of the activity observed using the control miRNA. (C) The inhibitory effect of *MIR155-3p* at the protein level of CREBRF and CREB3. We performed western blotting at 48 h after *MIR155-3p* inhibitor and mimic transfection into U251 and T98G cells and observed significantly decreased CREBRF protein levels in hypoxia-treated U251 and T98G cells. As CREBRF is a negative regulator of CREB3, the CREB3 level showed an opposite trend. (D) The *CREB3* siRNA knockdown decreases autophagy levels and ATG5 protein levels of U251 and T98G cells. (E) and (F) The key pathway proteins in primary glioma cells by western blotting. (G) The CREB3-induced autophagy-related (ATG) genes in U251 and T98G cells by quantitative real-time PCR. (H) *CREB3* siRNA inhibits GFP-LC3B translocation. pSELECT-GFP-LC3B-transfected U251 cells treated with IL6 (20 ng/ml) for 24 h. Scale bar: 50 μm. Quantitative analysis of GFP-LC3B puncta is shown in the right panel. The data shown are the mean ± s.d. of 4 independent experiments. * and ^#^, P < 0.05; ** and ^##^, P < 0.01; 2-tailed t test. 155i, *MIR155-3p* inhibitor; 155m, *MIR155-3p* mimic; NC, negative control.

Next, we searched for potential *MIR155-3p* target genes in miRDB (http://mirdb.org/miRDB/index.html) and identified the best potential target gene, CREBRF, with a full target score and 3 perfectly strong binding seed sequences (http://mirdb.org/cgi-bin/search.cgi?searchType=miRNA&full=mirbase&searchBox=MIMAT0004658). To assess the direct inhibitory effect of *MIR155-3p* on CREBRF gene transcription, 3′-UTR seed mutation and 3′-UTR luciferase assays were performed using U251 glioma cells (Fig. 8A). The luciferase activity in *MIR155-3p*-transfected cells decreased to approximately half of the activity observed using the control miRNA (Fig. 8B). To examine the inhibitory effect of *MIR155-3p* at the protein level, we performed western blotting at 48 h after *MIR155-3p* inhibitor and mimic transfection into U251 and T98G cells and observed significantly decreased CREBRF protein levels in hypoxia-treated U251 and T98G cells. The level of CREBRF protein was decreased significantly in *MIR155-3p* mimic-transfected glioma cells compared to those transfected with the negative control miRNA but increased significantly in *MIR155-3p* inhibitor-transfected glioma cells (Fig. 8C). As CREBRF is a negative regulator of CREB3, the CREB3 level showed an opposite trend (Fig. 8C).

To further test the relationship between the autophagy level and *MIR155-3p*-induced CREB3, we utilized a specific *CREB3* siRNA for knockdown. As expected, the cells transfected with *CREB3* siRNA exhibited a reduction in CREB3 protein and significantly reduced autophagy under conditions of both normoxia and hypoxia (Fig. 8D). To elucidate the mechanisms of CREB3-induced autophagy, several autophagy-related (*ATG*) genes were examined by quantitative real-time PCR in T98G and U251 cells, revealing a significant decrease in *ATG5* after CREB3 knockdown (Fig. 8G) that was confirmed by western blot in both cell lines (Fig. 8D). However, *ATG3*, which was only decreased in U251 cells, and other ATGs exhibited no change at the protein level (data not shown).

Furthermore, we confirmed the changes in those key pathway proteins in primary glioma cells by western blotting (Fig. 8E and F). In conclusion, it is tempting to postulate that the hypoxia-IL6-p-STAT3-*MIR155-3p*-CREBRF-CREB3-ATG5 pathway plays a pivotal role in hypoxia-induced autophagy in glioma cells (Fig. 12).

### Inhibition of IL6 induces autophagy-enhanced apoptosis in GBM cells

**Figure 9.**
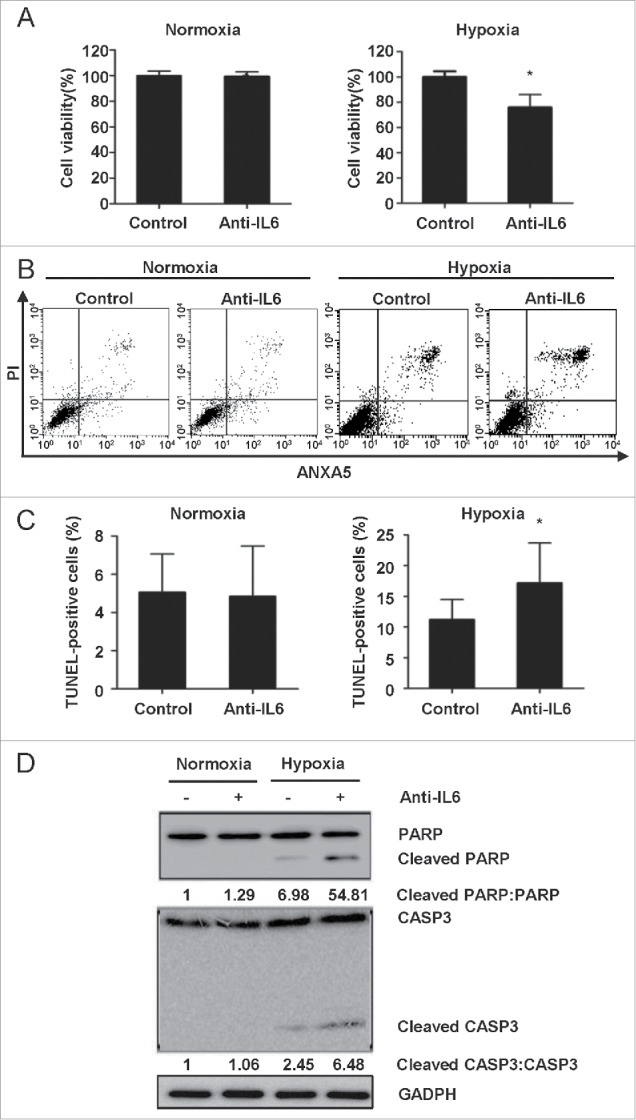
Inhibition of IL6 induces apoptosis in GBM cells. (A) A CCK-8 assay was performed to assess cell viability in hypoxia-treated U251 cells in the presence or absence of an IL6 antibody for 48 h. (B) and (C) ANXA5 FITC-PI and TUNEL staining assays were performed to examine the level of apoptosis in hypoxic GBM cells treated with an IL6 antibody (1 μg/ml). (D) Cleavage of PARP and CASP3 was induced by the IL6 antibody (1 μg/ml) in hypoxic GBM cells. PARP, cleaved PARP, CASP3 and cleaved CASP3 levels were examined by western blot analysis. GAPDH served as the loading control. The data are the mean ± s.d. *, P < 0.01 compared with the control group.

In recent years, accumulating evidence has demonstrated that autophagy may function as a protective mechanism in tumor cells and that therapy-induced apoptosis can be potentiated by autophagy inhibition.^53,54^ To determine the biological significance of autocrine and paracrine IL6-induced autophagy on apoptotic cell death, the recombinant human IL6 antibody was utilized to prevent autophagy through antibody neutralization. A CCK-8 assay was utilized to determine cell viability, and ANXA5#x2011;FITC-PI and TUNEL staining assays were performed to examine the level of apoptosis in GBM cells. As shown in Figure 9A, the IL6 antibody significantly suppressed GBM cells only under hypoxic conditions. In agreement with this observation, hypoxia-induced apoptotic cell death was augmented in the presence of the IL6 antibody, as demonstrated by ANXA5–x2011;FITC-PtdIns (Fig. 9B) and TUNEL (Fig. 9C) assays. During apoptosis, CASP3, as the final effector molecule, cleaves PARP into fragments of 85 kDa and 25 kDa, and PARP cleavage is one of the most common characteristics of apoptosis.^55^ Accordingly, we examined cleaved CASP3 and PARP protein levels by western blot analysis, and the data further indicated that application of the IL6 antibody resulted in the activation of apoptotic signaling pathways, including activated CASP3 and PARP (Fig. 9D). This type of crosstalk between autophagy and apoptosis provides a possible avenue for interference via hypoxia-induced autophagy of glioma cells through anti-IL6 adjuvant therapy in the treatment of glioma patients.

### Tocilizumab inhibits tumor growth by antagonizing hypoxia-induced autophagy and promoting apoptosis in a xenograft tumor model

**Figure 10.**
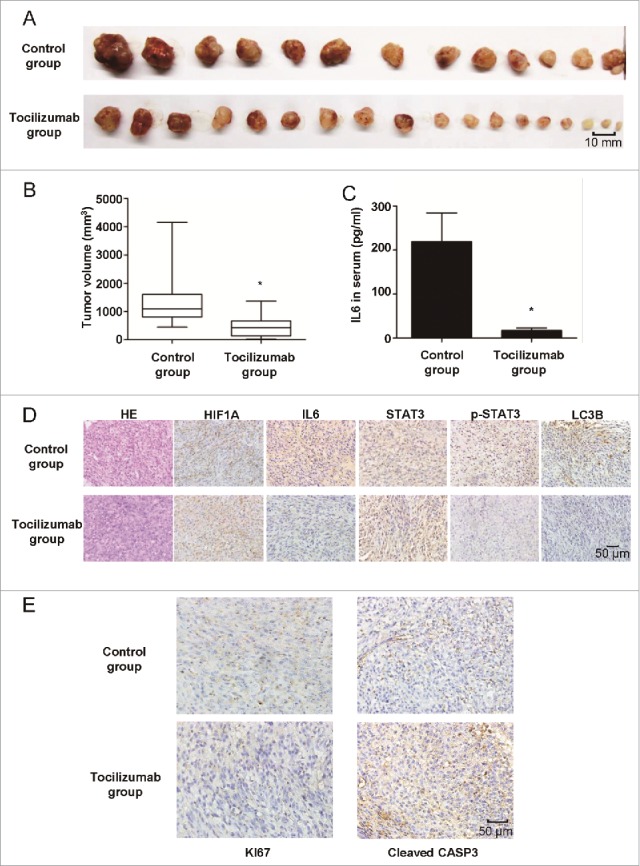
The IL6 monoclonal antibody tocilizumab inhibits the growth of GBM cells in vivo by inhibiting IL6-induced autophagy and promoting apoptosis. (A) and (B) Tocilizumab markedly inhibited tumor growth in U251 cell xenografts, as measured by tumor volume. *, P < 0.001 compared with the control group. (**C**) IL6 in the peripheral blood serum of the tocilizumab group was maintained at a lower level, as examined by ELISA, relative to the control group. *, P < 0.001 compared with the control group. (D) Tocilizumab reduced the expression of IL6, p-STAT3 and LC3B in all xenograft specimens, as evaluated by immunohistochemical staining. (E) Tocilizumab inhibited the proliferation and induced apoptosis in xenografts. The expression of KI67 and cleaved CASP3 in all xenograft specimens was examined by immunohistochemical staining.

Tocilizumab, a novel monoclonal antibody from Roche, recently became available in China and is mainly used in the treatment of rheumatoid arthritis due to its targeting of IL6 receptors. As discussed above, we found that the IL6 antibody exerted a suppressive effect on glioma cells in vitro by antagonizing hypoxia-induced autophagy and promoting apoptosis, suggesting that tocilizumab may inhibit glioma growth in vivo. Therefore, we injected tocilizumab into the caudal veins of nude mice that bore subcutaneous U251 xenografts. No major side effects were noted throughout the study. At the end of the study, we harvested the subcutaneous tumors and peripheral blood serum for analysis. Relative to the control group, the average tumor volumes of the tocilizumab group were significantly decreased (Fig. 10A and B), and IL6 in the peripheral blood serum was lower according to ELISA (Fig. 10C). To examine whether the treatment mechanism agreed with our hypothesis, we used immunohistochemical staining to detect the expression of IL6, STAT3 and p-STAT3, LC3B, HIF1A, KI67 and cleaved CASP3 in the xenograft specimens. As shown by HE staining (Fig. 10D), the U251 xenografts grew in a more compact manner and were more homogeneous compared with glioma specimens; however, IL6, STAT3 and p-STAT3, LC3B, and HIF1A staining was essentially similar. As expected, the expression levels of IL6 in serum and IL6, p-STAT3 and LC3B in xenograft tissue were significantly decreased in the tocilizumab group (Fig. 10D). Finally, a pronounced decrease in tumor cell proliferation (KI67) and an increase in apoptosis (cleaved CASP3) were noted in the tocilizumab-treated xenografts (Fig. 10E). These findings suggested an unexpected role for anti-IL6 therapy in the treatment of glioma by blocking autophagy and inducing apoptosis in glioma cells.

**Figure 11.**
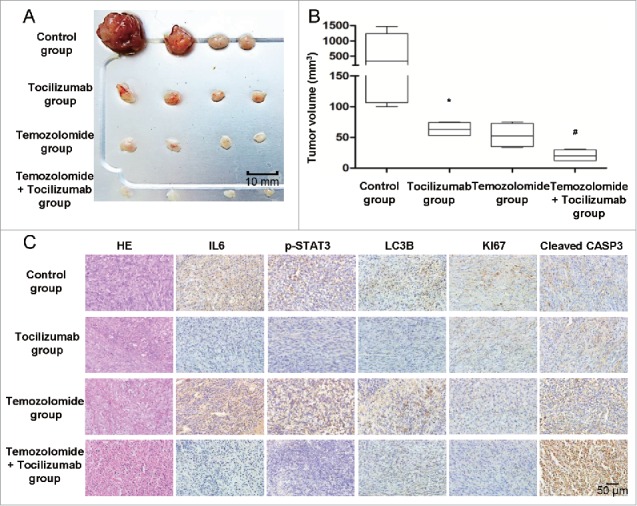
The IL6 monoclonal antibody tocilizumab increases the efficiency of temozolomide for GBM treatment in vivo by inhibiting IL6-induced autophagy and promoting apoptosis. (A) and (B) Tocilizumab and temozolomide combination therapy markedly inhibited tumor growth in U251 cell xenografts, as measured by tumor volume. *, P < 0.05 compared with the control group. ^#^, P < 0.05 compared with the temozolomide group. (C) Tocilizumab reduced the expression of IL6, p-STAT3 and LC3B and induced apoptosis in temozolomide-treated xenograft specimens, as examined by immunohistochemical staining.

It is well known that autophagy promotes the survival of tumor cells exposed to chemotherapy. However, there are different opinions concerning chemotherapy-induced autophagy and the efficiency of therapy.^56,57^ Furthermore, it remains unclear whether blockage of chemotherapy-induced autophagy could increase treatment efficiency. Temozolomide (TMZ), an oral chemotherapy drug, is used in the treatment of some brain tumors, as a second-line treatment for astrocytoma and as a first-line treatment for glioblastoma.^58^ In the present study, we investigated the combination therapy of temozolomide and tocilizumab in our xenograft tumor models and subsequently examined the ability of anti-IL6 therapy to reduce temozolomide-induced autophagy and improve its efficiency in vivo. As anticipated, the combination treatment resulted in a reduction in tumor volume compared with the single-drug groups (Fig. 11A and B). We then detected the expression of IL6, p-STAT3, LC3B, KI67 and cleaved CASP3 in the xenograft specimens by immunohistochemical staining. As expected, xenograft expression of IL6, p-STAT3 and LC3B in the temozolomide group was significantly increased (Fig. 11C), but combination therapy with tocilizumab blocked chemotherapy-induced autophagy and increased treatment efficiency by inducing significant apoptosis (Fig. 11C). To gain further insight into the involvement of the IL6-p-STAT3-*MIR155-3p* pathway in autophagy in vivo, we tested the effects of IL6 and *MIR155-3p* knockdown on tumor growth. To verify whether IL6 and *MIR155-3p* knockdown in glioma cells could block autophagy and induce apoptosis in vivo, adenovirus vector-transfected U251 cells were transplanted into nude mice through subcutaneous injection. The results indicated that knockdown of IL6 and *MIR155-3p* could inhibit tumor growth in vivo by blocking autophagy and inducing apoptosis in glioma cells, confirming the central role played by the IL6-p-STAT3-*MIR155-3p* pathway in controlling hypoxia-induced autophagy (Fig. S4).

**Figure 12.**
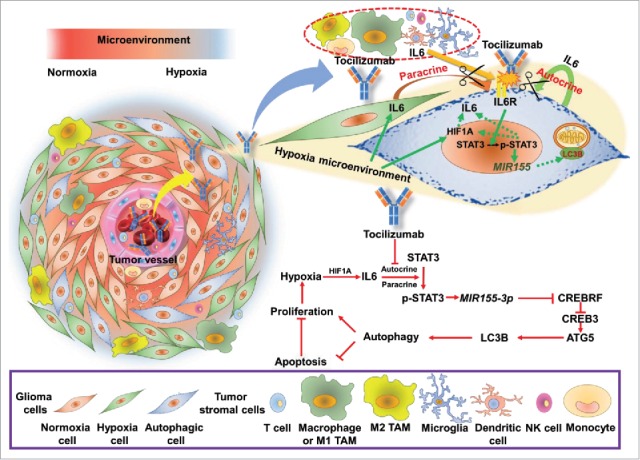
Proposed model and hypothesis of the novel tumor-promoting mechanism of IL6 and the therapeutic efficacy of tocilizumab: hypoxia-induced IL6 is a potent autophagy initiator in glioblastoma via the p-STAT3-*MIR155-3p*-CREBRF pathway. The hypoxic regions around the tumor vessels (Fig. 1B) and hypoxia-induced IL6 trigger hypoxic glioma cell autophagy through the p-STAT3-*MIR155-3p*-CREBRF pathway. Because upregulated autophagy inhibits tumor apoptosis and promotes proliferation, we blocked the IL6 receptor using tocilizumab and significantly suppressed tumor growth by shifting from autophagy to apoptosis. TAM, tumor-associated macrophage.

These data recapitulated the in vitro observations and showed that anti-IL6 therapy could be utilized for the treatment of glioma patients by antagonizing hypoxia-induced autophagy and promoting the apoptosis of tumor cells in hypoxic regions (Fig. 12).

## Discussion

Autophagy promotes tumor survival under hypoxic metabolic stress conditions and mediates resistance to anti-tumor therapies such as radiation and chemotherapy.^59,60^ Although numerous anti-tumor agents have been reported to trigger cellular autophagy,^61^ novel adjuvant therapeutic strategies that efficaciously target GBMs are desperately needed to improve the currently unfavorable outcome of glioma patients. Recent accumulating evidence has demonstrated that the inhibition of autophagy triggers apoptosis.^47^ In the present study, we discovered a novel anti-autophagy therapeutic strategy by targeting hypoxia-induced IL6 in glioma cells. Our hypothesis that hypoxia-induced IL6 is a potent autophagy initiator in GBM via the p-STAT3-*MIR155-3p* pathway was confirmed by a series of experiments in vitro and in vivo. As shown in Fig. 10, hypoxic regions were distributed around tumor vessels (Fig. 1B), and hypoxia-induced IL6 triggered autophagy in hypoxic glioma cells through the p-STAT3-*MIR155-3p* pathway. Because upregulated autophagy inhibits tumor apoptosis and promotes proliferation, we blocked the IL6 receptor using tocilizumab, which significantly suppressed tumor growth by shifting from autophagy into apoptosis (Figs. 9 and 10E). This work is significant because it characterizes the pivotal role of the IL6-p-STAT3-*MIR155-3p* pathway in hypoxia-induced autophagy in glioma cells, and this is the first demonstration of the therapeutic efficacy of IL6 receptor monoclonal antibody injection for the treatment of gliomas in vivo.

The tumor microenvironment plays a critical role in tumor progression, and malignant proliferative tumor cells are deprived of oxygen as the tumor rapidly outgrows its blood supply.^62^ Tumor hypoxia can strongly induce cells to develop an aggressive and treatment-resistant phenotype that leads to rapid progression and poor prognosis.^16^ HIF1A, a key transcription factor involved in adaptation to hypoxic stress,^63^ plays an important role in hypoxia-induced autophagy, activating several autophagy-inducing molecules, such as BNIP3 (BCL2/adenovirus E1B 19kDa interacting protein 3) and IGFBP3 (insulin like growth factor binding protein 3).^45,64^ In addition, HIF1A-independent AMPK activates more severe hypoxia,^22^ and AMPK can contribute to autophagy via an MTOR inhibition mechanism.^65,66^ Nevertheless, similar to our previous demonstrations of high IL6 expression levels in gliomas and many other tumors,^9-11,14,15,67^ we found in the current study that IL6 also plays a role in hypoxia-induced autophagy.

IL6, a biomarker that indicates poor prognosis when overexpressed in many malignant tumors,^9-13^ promotes tumor progression by autocrine and paracrine mechanisms and binds to its receptor to activate downstream STAT3.^34^ Both phosphorylated and nonphosphorylated STAT3 can positively regulate the expression of IL6 and further promote a feed-forward loop.^68-70^ Furthermore, IL6 is a cytokine that is not only involved in inflammation and infection responses but also in the regulation of metabolic, regenerative, and neural processes as well as tumor progression.^71^ There are several lines of evidence suggesting that IL6 is a pleiotropic cytokine with significant functions in regulating the immune system.^72^ For instance, as a potent pro-inflammatory cytokine, IL6 plays a pivotal role in host defense against pathogens and acute stress. However, increased or deregulated expression of IL6 significantly contributes to the pathogenesis of various human diseases, including tumors.^72^ IL6R (interleukin 6 receptor) exists in 2 forms, a membrane-bound and soluble form;^73^ after binding IL6, the IL6-IL6R complex activates JAK1 (Janus kinase 1), JAK2 (Janus kinase 2), and TYK2 (tyrosine kinase 2) via IL6ST/gp130 (interleukin 6 signal transducer) signaling, leading to the phosphorylation of STAT3. STAT3 is an important regulator of a number of anti-apoptotic genes, and its activity is associated with tumor growth, survival, angiogenesis, and metastatic processes,^74^ especially in glioblastoma progression.^33^ However, the review articles cited have only summarized the current role of IL6-STAT3-regulated processes in promoting GBM progression with regard to proliferation, migration,^75^ anti-apoptosis, angiogenesis,^76^ relapse, and immune escape,^77,78^ but without the important aspect of autophagy. However, recent reports have shown that STAT3 affects autophagy via the transcriptional regulation of several autophagy-related genes, such as *HIF1A* and other genes, and microRNAs with autophagy-modulating targets.^79^ Our study first clearly demonstrated that the hypoxia-IL6-STAT3-*MIR155-3p*-CREBRF-CREB3 pathway mediates IL6-induced autophagy in glioma cells under hypoxic conditions. These observations reveal a novel positive feedback mechanism of the IL6-STAT3-HIF1A pathway that is promoted by hypoxia-induced autophagy and provide new insight into the contribution of IL6 to tumor progression by initiating protective autophagy. Because multiple upstream activators converge on the STAT3 pathway, targeting only one of these activators of the STAT3 pathway is unlikely to be sufficient for therapy. For tumor types that express high levels of IL6, such as GBM,^80^ our in vivo experimental results suggest that tocilizumab may be a potential therapy for glioma patients, at least as an adjuvant therapy combined with current chemotherapy.

Membrane-bound IL6R stimulates a classical signaling pathway, whereas soluble IL6R acts in a trans-signaling pathway.^75^ The classical and trans-signaling pathways are thought to contribute to the anti-inflammatory and pro-inflammatory activities of IL6, respectively,^72^ and the proliferation and survival of tumor cells are promoted by IL6 through both paracrine and autocrine mechanisms. After activation by IL6, various types of cells, such as tumor-associated macrophages,^81^ myeloid-derived suppressor cells,^77,78^ and endothelial cells,^72^ are involved in the development of a suppressive immunomicroenvironment and a metastatic tumor microenvironment.^82^ Several IL6-associated cytokines have growth factor activity, and inflammatory mediators such as HMGB1, IL23, and IL17 can promote tumor growth by activating the IL6-STAT3 pathway in a mouse model of melanoma.^83^ In addition, IL6 modulates the balance between regulatory T cells and T helper 17 cells in the tumor microenvironment and promotes the generation of cancer stem cells from noncancer stem cells.^84^

miRNAs are increasingly reported to be involved in the modulation of different autophagic stages.^37^ For example, ULK1 (unc-51 like autophagy activating kinase 1) is targeted by *MIR20A* and *MIR106*;^85^ BECN1/beclin 1 by *MIR30A, MIR376B* and *MIR519A*;^86-89^ RAB5A (RAB5A, member RAS oncogene family) by *MIR101* and *MIR630*;^89,90^ RB1CC1/FIP200 (RB1 inducible coiled-coil 1) by *MIR224*;^91^ and ATGs by *MIR30A, MIR181A, MIR374A, MIR630, MIR376B, MIR204, MIR224, MIR375, MIR519A, MIR885*, and *MIR-101*.^91-93^ Almost all of these miRNAs are negative regulators of autophagy. *MIR155-5p* and *MIR7* are rare miRNA inducers of autophagy.^94^ Mature miRNAs are processed from longer 70-nucleotide pre-miRNAs by RNASE3-like enzymes in the cytoplasm; pre-miRNAs are long duplex hairpins that contain several bulges or mispairings and are capped by an apical loop of variable size.^95,96^ Previous studies have suggested that the 5′ arm of the pre-miRNA stem-loop sequence will become the unique, mature miRNA, whereas the 3′ arm will be rapidly degraded.^97^ Nevertheless, growing evidence now suggests that miRNAs encoded from the 3′ arm (−3p) exist simultaneously with those encoded by the 5′ arm (−5p) and target a completely different set of mRNAs because of their disparate sequences, even though they originate from the same precursor.^98^ Although *MIR155-5p* has been previously reported to be an effective inducer of autophagy by directly targeting RHEB, RICTOR, and RPS6KB2 of the MTOR pathway in CNE and HeLa cells,^37^ *MIR155-3p*, a completely different mature miRNA of the *MIR155* pre-miRNA, is also produced, though its exact role in tumors has not yet been elucidated. Our data first identified *MIR155-3p*, which was found in a screen of our hypoxia microarray, as a central player in the hypoxia-induced IL6 pro-autophagic pathway. The direct regulation of *MIR155* pre-miRNAs by STAT3 has also been confirmed by Li et al.^99^ Unlike *MIR155-5p*, the TargetScan 6.2 algorithm cannot currently be utilized to determine the putative targets of *MIR155-3p*. However, one would speculate that this miRNA could potentially regulate the expression of thousands of mRNA targets.^100^ Nonetheless, the miRDB database can be used for *MIR155-3p* target prediction, and we found 248 potential targets of *MIR155-3p*. Our results indicated that the top predicted target, CREBRF, is a direct target of *MIR155-3p* and mediates the autophagic-promoting effect of *MIR155-3p* by regulating CREB3 and, in turn, ATG5. Therefore, our theoretical hypoxia-IL6-p-STAT3-*MIR155-3p*-autophagy pathway appears to play crucial roles in GBM malignant progression, and STAT3, which is centrally located, may have feed-forward control and transcriptionally regulate *MIR155-3p* (Fig. 12).

More than a half a century after its discovery, novel roles for the IL6 signaling pathway in tumor formation and progression have been consistently uncovered, including roles in regulating the proliferation, migration, activation, morphology, and metabolic state of tumor cells. However, in preclinical models of wide metastatic disease, IL6 inhibitors do not lead to disease regression.^101^ Tocilizumab has recently been approved for the treatment of rheumatoid arthritis by the FDA and China's SFDA, but there are no apparent plans to use this agent for malignancies. Tocilizumab treatment in patients with rheumatoid arthritis results in better clinical outcomes,^102,103^ and other related studies have shown that serum IL6 levels and serum MIF (macrophage migration inhibitory factor [glycosylation-inhibiting factor]) levels are significantly decreased with tocilizumab treatment.^104,105^ Regardless, our in vivo findings in a GBM mouse model provide insight that may be useful for the development of efficacious anti-IL6 therapies by tocilizumab injection. There are at least 2 possible explanations for this unexpected therapeutic effect on gliomas: the unique biological characteristics of glioma cells mentioned above and, most importantly, the unique immune microenvironment of the tumor. In addition, the decreased serum MIF level after tocilizumab injection might be another reason due to the tumor-promoting effects of MIF in gliomas.^106,107^

It is well established that the central nervous system is not devoid of immune cells and does not possess only ineffective immune cells; instead, immunosuppressive regulation of the immune balance occurs in gliomas,^108-110^ similar to that which occurs in the regulation of HLA-related molecules,^111-114^ the secretion of immunosuppressive factors, and the recruitment of immunosuppressive cells.^115^ The population of type M2 glioma-associated macrophages (GAMs) is thought to largely contribute to the induction of the immunosuppressive glioma microenvironment. Multiple immunosuppressive factors are secreted by glioma cells, including IL6, which could polarize GAMs toward tumor-promoting M2 GAMs.^116^ In contrast, M2 GAMs deteriorate this microenvironment by releasing IL6,^117^ which also stimulates glioma growth, neovascularization, and invasiveness.^117,118^ Furthermore, myeloid-derived suppressor cells are another remarkable immunosuppressive cell type in gliomas that are also induced by tumor-derived IL6.^119^ Although the subcutaneous gliomas in our studies are substantially different than in situ tumors, we detected an abundance of M2 tumor associated macrophage infiltration via immunohistochemical assays. Therefore, the observed therapeutic effect of tocilizumab may partially be attributed to blockade of the immunosuppressive effects of IL6.

In conclusion, this study demonstrated that hypoxia-induced IL6 is a potent autophagy inducer in GBM via the p-STAT3-*MIR155-3p*-CREBRF-CREB3-ATG5 pathway and that IL6-induced autophagy could protect GBM cells from apoptotic death. We investigated the ability of tocilizumab to block this pathway at the source, and our positive results suggest potential uses of this drug for glioma patients in anti-IL6 therapeutic strategies, such as adjuvant therapy combined with TMZ. However, modifications of the immunosuppressive microenvironment in the presence of tocilizumab remain to be defined, and evaluation of the therapeutic efficiency of tocilizumab combined with other common strategies would be helpful for advancing clinical investigations.

## Materials and methods

### Tissue samples and cell lines

The human glioma cell lines U251 and T98G were purchased from the Chinese Academy of Sciences Cell Bank and identified by the STR site detection assay (Microread Genetics Co, Beijing). One hundred and one human glioma tissue samples including 44 low-grade gliomas (8 grade I tumors and 36 grade II tumors), 57 high-grade gliomas (21 grade III tumors and 36 grade IV tumors), and 3 normal brain tissues from decompression operation were obtained from the Department of Neurosurgery of Qilu Hospital of Shandong University from October 2013 to June 2015. The glioma specimens were verified and classified according to the WHO classification standard of tumors by 2 experienced clinical pathologists. Our study was approved by the Institutional Review Board of Shandong University. Written informed consent was obtained from all patients, and the hospital ethics committee approved the experiments.

### Reagents, cell culture and hypoxia treatment

All reagents in the study are as follows: 3-methyladenine (ApexBio Technology, A8353), bafilomycin A_1_ (Enzo Life Sciences, BML-CM110-0100), recombinant human IL6 (ProSpec, CYT-213), anti-human IL6 (ProSpec, ANT-109), tocilizumab/Actemra® (Roche Pharma, S20130020), temozolomide (Sigma-Aldrich, T2577). All cells were cultured in DMEM (Invitrogen, SP10001) supplemented with 10% fetal bovine serum (Gibco, 10099-141) and maintained at 37°C with 5% CO_2_ in a humidified chamber. Hypoxic conditions were induced by incubating the cells in a modular incubator chamber flushed with a gas mixture containing 1% O_2_, 5% CO_2_, and 94% N_2_ at 37°C.

### Immunohistochemical staining

Human glioma tissue samples or solid tumors removed from sacrificed mice were fixed with 4% formaldehyde. Paraffin-embedded tumor tissues were sectioned to 5-μm thickness and mounted on positively charged microscope slides, and 1 mM EDTA (pH 8.0; Beyotime Biotechnology, P0085) for HIF1A or citrate solution (pH 6.0; Beyotime Biotechnology, P0083) for other antigens was used for antigen retrieval. Endogenous peroxidase activity was quenched by incubating the slides in methanol containing 3% hydrogen peroxide, followed by washing in phosphate-buffered saline (PBS; Beyotime Biotechnology, ST476) for 6 min. The sections were incubated for 2 h at room temperature with normal goat serum (ZSGB-BIO, ZLI-9021) and subsequently incubated at 4°C overnight with primary antibodies (1:200, HIF1A [Abcam, ab82832]; 1:400, IL6 [Abcam, ab6672, ab9324]; 1:300, STAT3 [Abcam, ab5073]; 1:100, p-STAT3 [Abcam, ab76315]; 1:400, LC3B [Abcam, ab48394]; 1:100, KI67 [Cell Signaling Technology, 9027]; and 1:300, cleaved CASP3 [Cell Signaling Technology, 9664]). The sections were then rinsed with PBS and incubated with horseradish peroxidase-conjugated goat anti-rabbit or anti-mouse antibodies (ZSGB-BIO, PV-9000), followed by reaction with diaminobenzidine (ZSGB-BIO, ZLI-9033) and counterstaining with Mayer's hematoxylin (Beyotime Biotechnology, C0107). HE staining was performed free of charge by the pathology department of Qilu Hospital of Shandong University. Evaluation of the staining reaction was performed in accordance with the immunoreactive score = staining intensity × percentage of positive cells.^120,121^ A staining intensity value of 0 was negative; 1, weak; 2, moderate; and 3, strong. A percentage of positive cells value of 0 was negative; 1, 10% positive cells; 2, 11-50% positive cells; 3, 51-80% positive cells; and 4, more than 80% positive cells. Five visual fields from different areas of each tumor were used for the immunoreactive score evaluation.

### Immunofluorescence staining

U251 cells were plated on glass slides in 24-well culture plates at a concentration of 2×10^5^ cells/well for 24 h and subsequently treated with reagents for an additional 48 h in serum-free DMEM. Thereafter, the cells were fixed with a 4% formaldehyde solution in PBS, permeabilized with 0.5% Triton X-100 (Beyotime Biotechnology, ST795) in PBS, stained with the primary antibody overnight, and labeled with anti-mouse or anti-rabbit IgG conjugated with FITC (Santa Cruz Biotechnology, sc-358949, sc-2012). The cells were counterstained with DAPI and observed under an Olympus BX61 fluorescence microscope. Images were scanned using a DP71 CCD (charge-coupled device) digital camera.

### GFP-LC3 stable cell lines and quantitative GFP-LC3 analyses

A U251 GFP-LC3 stable cell line was established by transient transfection of the pSELECT-GFP-LC3 lentiviral vector (Invivogen, VXI0541). GFP-LC3 puncta formation under normoxic or hypoxic conditions was determined by capturing images using a DP71 CCD digital camera microscope (Olympus). To quantify autophagic cells after drug treatment, we counted the number of autophagic cells as determined by the presence of GFP-LC3 puncta (≥20 puncta = a positive cell) in 100 high-power fields.

### Western blot analysis

After the desired treatment, cells were washed twice with cold PBS and harvested with a rubber scraper. Cell pellets were lysed and kept on ice for at least 30 min in a buffer (Beyotime Biotechnology, P0013) containing 50 mM Tris-HCl, pH 7.4, 150 mM NaCl, 0.5% Nonidet P-40 (Beyotime Biotechnology, P0013F), 50 mM NaF, 1 mM Na_3_VO_4_, 1 mM phenylmethylsulfonyl fluoride (Beyotime Biotechnology, ST506). The lysates were cleared by centrifugation, and the supernatant fractions were collected. Cell lysates were then separated by SDS-PAGE (Beyotime Biotechnology, P0012A) and subjected to western blot analysis with primary antibodies and horseradish peroxidase-conjugated secondary antibodies. Antibodies against the following were used for western blotting: SQSTM1 (Cell Signaling Technology, 5114S), LC3B (Abcam, ab63817), GAPDH (Cell Signaling Technology, 3683S), STAT3 (Abcam, ab68153), p-STAT3 (Abcam, ab76315), ACTB (Cell Signaling Technology, 12262S), PARP (Abcam, ab32138), cleaved PARP (Abcam, ab32064), CASP3 (Cell Signaling Technology, 9668T), cleaved CASP3 (Cell Signaling Technology, 9664S), KI67/PCNA (Cell Signaling Technology, 2586S), CREBRF (Santa Cruz Biotechnology, sc-133747), CREB3 (Abcam, ab42454), and ATG5 (Abcam, ab108327). GAPDH served as the loading control in all experiments involving hypoxic treatment due to the upregulation of ACTB under hypoxia.

### ELISA

GBM culture supernatant was obtained from plates of treated cells, and blood serum samples were collected during the in vivo experiment. ELISAs were processed using a Human IL6 Valukine ELISA Kit (R&D Systems, VAL102) according to the manufacturer's instructions. The absorbance at 450 nm was measured using a microplate reader.

### Transmission electron microscopy

Cells were fixed with 3% glutaraldehyde in PBS for 2 h, washed 5 times with 0.1 M cacodylate buffer, and postfixed with 1% OsO_4_ in 0.1 M cacodylate buffer containing 0.1% CaCl_2_ for 1.5 h at 4°C. The samples were then stained with 1% Millipore-filtered uranyl acetate, dehydrated in increasing concentrations of ethanol, infiltrated, and embedded in LX-112 medium (Ladd Research Industries, 21210). After polymerization of the resin at 60°C for 48 h, ultrathin sections were cut with a Leica Ultracut microtome (Leica). Sections were stained with 4% uranyl acetate and lead citrate, and images were obtained using a JEM-100cxII electron microscope (JEM).

### Small interfering RNA, miR inhibitor/mimics and adenovirus vector transfection

IL6 and negative control siRNAs were synthesized by GenePharma (China). The *MIR155-3p* inhibitor/mimics and negative control sequences were synthesized by Rio-Bio (China). The *MIR155-3p* inhibitor, IL6 siRNA, CREB3 siRNA and negative control adenovirus vectors were also synthesized by Rio-Bio (China). The sequences of siRNAs were as follows: Human IL6-207, 5′-CCC AGG AGA AGA UUC CAA ATT-3′ (sense) and 5′-TTG GGU CCU CUU CUA AGG UUU-3′ (antisense); IL6-321, 5′-GGA GAC AUG UAA CAA GAG UTT-3′ (sense) and 5′-TTC CUC UGU ACA UUG UUC UCA-3′ (antisense); IL6-417, 5′-CUU CCA AUC UGG AUU CAA UTT-3′ (sense) and 5′-TTG AAG GUU AGA CCU AAG UUA-3′ (antisense); CREB3-1299, 5′-GCA GUC AGA AGU GCC GAA ATT-3′ (sense) and 5′-TTC GUC AGU CUU CAC GGC UUU-3′ (antisense). The sequences of *MIR155-3p* inhibitor/mimics were as follows: *MIR155-3p* inhibitor, 5′- UGU UAA UGC UAA UAU GUA GGA G −3′; *MIR155-3p* mimics, 5′- CUC CUA CAU AUU AGC AUU AAC A −3′ (sense) and 5′-UUA AUG CUA AUA UGU AGG AGU U-3′ (antisense). The sequences of negative control were as follows: 5′-UUC UCC GAA CGU GUC ACG UTT-3′ (sense) and 5′-ACG UGA CAC GUU CGG AGA ATT-3′ (antisense). The miRNA inhibitor negative control was as follows: 5′-CAG UAC UUU UGU GUA GUA CAA-3′. The siRNAs or inhibitor/mimics were transfected into U251 and T98G cells for 48 h using Lipofectamine 2000 (Invitrogen, 11668-027) according to the protocol of the manufacturer. The *MIR155-3p* inhibitor, IL6 siRNA and negative control adenovirus vectors were transfected into U251 cells for 72 h according to the protocol of the manufacturer.

### Cell viability assay

The CCK-8 assay (Dojindo Molecular Technologies, CK04) was used to test cell viability. Tumor cells in medium containing 10% fetal bovine serum were seeded into 96-well, flat-bottomed plates at 5×10^3^ cells/well and incubated at 37°C overnight. After the desired treatment, the cells were incubated for an additional 4 h with 100 μl of serum-free DMEM and 10 μl of CCK-8 at 37°C. The absorbance at 450 nm was measured using a microplate reader.

### ANXA5-fluorescein isothiocyanate (FITC) assay

To assess the degree of apoptosis of different groups of GBM cells, the extent of ANXA5-FITC-propidium iodide (PI) staining was determined by flow cytometry using the ANXA5/PtdIns staining kit (Beyotime Biotechnology, C1063). Samples were measured using an Epics XL-MCL flow cytometer (Beckman Coulter, Brea, CA, USA) and analyzed with WinMDI 2.8 software.

### TUNEL assay

GBM cells were plated on glass slides in 24-well culture plates at 2×10^5^ cells/well for 24 h and subsequently treated with drugs for an additional 48 h in serum-free DMEM. Glass slides with GBM cells and paraffin-embedded tumor sections were stained by the TUNEL technique using a TACS®2 TdT-Fluor in situ apoptosis detection kit (Trevigen, 4822-96-K) according to the instructions of the manufacturer. TUNEL-positive cells were counted from at least 100 random fields under a fluorescence microscope.

### Human miRCURY™ LNA Array analysis

U251 cells were cultured under normoxic and hypoxic conditions (3 samples were prepared for each condition to serve as biological replicates), and then total RNA was extracted for microarray analysis. Total RNA was harvested using TRIzol (Invitrogen, IS-10007) and the miRNeasy mini kit (QIAGEN, 217004) according to the manufacturers' instructions. The RNA quantity was measured using a NanoDrop 1000, and the samples were labeled with the miRCURY™ Hy3™/Hy5™ Power labeling kit and hybridized onto the miRCURY™ LNA Array (v.18.0). After washing, the slides were scanned using the Axon GenePix 4000B microarray scanner. Scanned images were then imported into GenePix Pro 6.0 software (Axon) for grid alignment and data extraction. After normalization, significantly differentially expressed miRNAs were identified through Volcano Plot filtering. Finally, hierarchical clustering was performed to assess distinguishable gene expression profiles among samples.

### RNA extraction and real-time quantitative PCR

Total RNA was extracted using TRIzol according to the manufacturer's protocol. Then, total RNA (50 ng) was reverse-transcribed with miR stem-loop RT primers or with U6 RT primers using a ReverTra Ace qPCR RT kit (TOYOBO, FSQ-101) according to the manufacturer's protocol to generate cDNA. Real-time PCR was performed using a SYBR Premix Ex Taq™ Kit (TAKARA, RR420A) with each primer. The reactions were performed using a LightCycler 2.0 Instrument (Roche). U6 expression was used as the endogenous control. The absolute expression levels were calculated as concentration ratios using a Roche LightCycler® 2.0 system.

### Bioinformatic prediction and luciferase reporter assay

Common *MIR155-3p* targets predicted by computer-aided algorithms were obtained using the online miRNA prediction tool of miRBase. The reporter constructs containing pGL3-CREBRF and pGL3-mutCREBRF, with a mutated target seed sequence, were obtained from Bio-Asia (Jinan, China). Mutation of the p-STAT3 binding element (Bio-Asia, Jinan, China) and luciferase reporter assays were also performed using U251 glioma cells, which were cotransfected with luciferase reporters along with test substances or vehicle using Lipofectamine 2000. Forty-eight h after the cells were transfected, luciferase assays were performed using a luciferase assay kit according to the manufacturer's instructions.

### Tumor xenograft model

The experiments conformed to the Animal Management Rule of the Chinese Ministry of Health (documentation 55, 2001), and the experimental protocol was approved by the Animal Care and Use Committee of Shandong University. Fifty BALB/c nude (nu/nu) female mice were purchased from Vital River Laboratories in batches. U251 cells (5×10^6^ cells in 50 μl of serum-free DMEM) were inoculated subcutaneously into the right groin of the first batch of 5 5-wk-old female mice after acclimatization for a wk. Tumor volumes were calculated as (L ×W2)/2, where L is the length in millimeters and W is the width in millimeters. After 30 d, 3 of these mice developed a visible tumor mass, and the maximal tumor was resected using aseptic technique. The tumor tissue was rapidly cut into pieces of the same size, and each was transplanted subcutaneously into the right groin of a second batch of 45 female mice. When the tumors reached a mean volume of 90-120 mm^3^ (only 35 mice developed uniform tumors), the animals were randomized into 2 groups (15 in the control group and 20 in the treatment group). In vivo experiments were then performed to investigate the effect of tocilizumab injection: tocilizumab was injected into the caudal vein (8 mg/kg/month in normal saline), and an equivalent volume of normal saline was injected into the control group. Tumors were dissected at 30 d after transplantation and frozen in liquid nitrogen or fixed in formalin (2 mice were lost from each group due to suffocation), and blood serum samples were collected. Combination therapy tests of temozolomide plus tocilizumab were performed with the same xenograft tumor models. In the experiment, 20 mice were randomly divided into 4 groups. In the tocilizumab and combination groups, tocilizumab was injected into the caudal veins of 5 mice (8 mg/kg/mo in normal saline), with an equivalent volume of normal saline injected into 5 mice of the control and temozolomide groups. Intragastric administration of temozolomide (50 mg/kg) for 5 d in the temozolomide and combination groups was performed at the same time. Tumors were dissected at 30 d after transplantation and frozen in liquid nitrogen or fixed in formalin (1 mouse was lost from each group due to suffocation during intragastric administration). For in vivo IL6 and *MIR155-3p* knockdown tests, adenovirus vectors were transfected into U251 cells, and the same concentration of cell suspension was transplanted into nude mice (45 mice in 3 groups) through subcutaneous injection. Tumors were dissected at 28 d after transplantation and frozen in liquid nitrogen or fixed in formalin (due to individual differences, the negative control group and *MIR155-3p* knockdown group both included 5 mice without xenografts, and the IL6 knockdown group included 4 mice without xenografts).

### Statistical analysis

Data analyses were conducted with SPSS 16.0 (SPSS, IL, USA) and GraphPad-Prism5 (GraphPad, CA, USA). Descriptive statistics including means ± SD, Student's *t* test, non-parametric Kruskal-Wallis tests for multiple comparison, the Mann-Whitney Test for 2-group comparison, Kaplan-Meier plots, log-rank tests, one-way ANOVAs, Pearson's correlation test and Spearman's correlation test were used to analyze significant differences; *, P < 0.05; **, P < 0.01; and ***, P < 0.001 were considered statistically significant.

## Supplementary Material

KAUP_A_1178446_Supplementary_material.zip

## Abbreviations

3-MA: 3-methyladenine
3′-UTR: 3′-untranslated region
AMPK: adenosine 5′-monophosphate-activated protein kinase
ATG3: autophagy-related 3
ATG5: autophagy-related 5
BAF: bafilomycin A_1_
CASP3: caspase 3
CCK-8: cell counting kit-8
CD: clusters of differentiation
CREB3: cAMP responsive element binding protein 3
CREBRF: CREB3 regulatory factor
ELISA: enzyme-linked immunosorbent assay
ER: endoplasmic reticulum
GAM: glioma-associated macrophage
GAPDH: glyceraldehyde-3-phosphate dehydrogenase
GBM: glioblastoma
GFP: green fluorescent protein
HIF1A: hypoxia inducible factor 1 α subunit
IL6: interleukin 6
IL6R: interleukin 6 receptor
JAK1: Janus kinase 1
M1: macrophages type 1
M2: macrophages type 2
MAP1LC3B/ LC3B: microtubule associated protein 1 light chain protein 3 β
MIF: macrophage migration inhibitory factor (glycosylation-inhibiting factor)
*MIR*/miR/miRNA: microRNA
MTOR: mechanistic target of rapamycin (serine/threonine kinase)
PARP: poly(ADP-ribose) polymerase; p-STAT3, phosphorylated signal transducer and activator of transcription 3
siRNA: small interfering RNA; SQSTM1/p62, sequestosome 1
STAT3: signal transducer and activator of transcription 3
TMZ: temozolomide
TUNEL: deoxynucleotidyl transferase-mediated dUTP nick-end labeling
WHO: world health organization
