# Supplementary material for: A novel tumor-promoting mechanism of IL6 and the therapeutic efficacy of tocilizumab: Hypoxia-induced IL6 is a potent autophagy initiator in glioblastoma via the p-STAT3-MIR155-3p-CREBRF pathway
Source: Autophagy. 2016 May 10;12(7):1129–52. doi: 10.1080/15548627.2016.1178446 (PMC4990999; doi:10.1080/15548627.2016.1178446)
Supplement: KAUP_A_1178446_Supplementary_material.zip [file kaup-12-07-1178446-s001.zip › KAUP_A_1178446 Supplementary material.docx]

| **Table 1** Demographic parameters of patients participating in the study | | | |
| --- | --- | --- | --- |
|  |  | No. of Patients | N% |
| Assessable |  |  |  |
|  | Glioma | 101 | 97.12% |
|  | Normal brain tissues | 3 | 2.88% |
| Gender |  |  |  |
|  | Male | 62 | 59.62% |
|  | Female | 42 | 40.38% |
| Age (years) |  |  |  |
|  | Median (range) | 45.19 (4~75) |  |
| Pathological type |  |  |  |
|  | Astrocytoma | 28 | 26.92% |
|  | Anaplastic astrocytoma | 16 | 15.38% |
|  | Pilocytic astrocytoma | 5 | 4.81% |
|  | Oligodendroglioma | 9 | 8.65% |
|  | Anaplastic oligodendroglioma | 5 | 4.81% |
|  | Glioblastoma | 36 | 34.61% |
|  | Dysembryoplastic neuroepithelial tumor | 2 | 1.92% |
|  | Normal brain tissues | 3 | 2.88% |
| WHO tumor grade at diagnosis |  |  |  |
|  | I | 8 | 7.69% |
|  | II | 36 | 34.62% |
|  | III | 21 | 20.19% |
|  | IV | 36 | 34.62% |
