# Supplementary figures and images for: A novel tumor-promoting mechanism of IL6 and the therapeutic efficacy of tocilizumab: Hypoxia-induced IL6 is a potent autophagy initiator in glioblastoma via the p-STAT3-MIR155-3p-CREBRF pathway
Source: Autophagy. 2016 May 10;12(7):1129–52. doi: 10.1080/15548627.2016.1178446 (PMC4990999; doi:10.1080/15548627.2016.1178446)

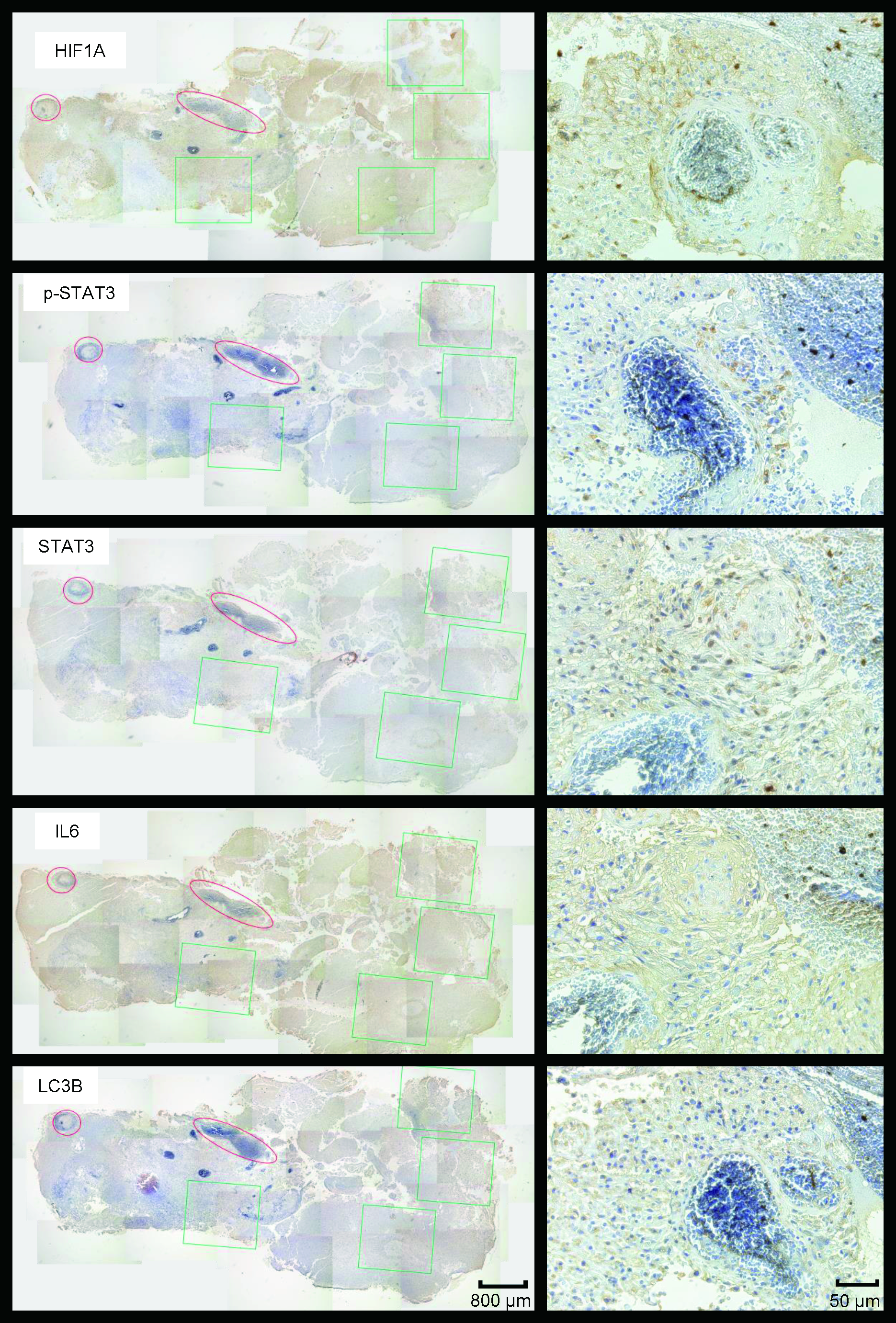

Supplement: KAUP_A_1178446_Supplementary_material.zip [file kaup-12-07-1178446-s001.zip › 2015AUTO0429R2-s02.tif]

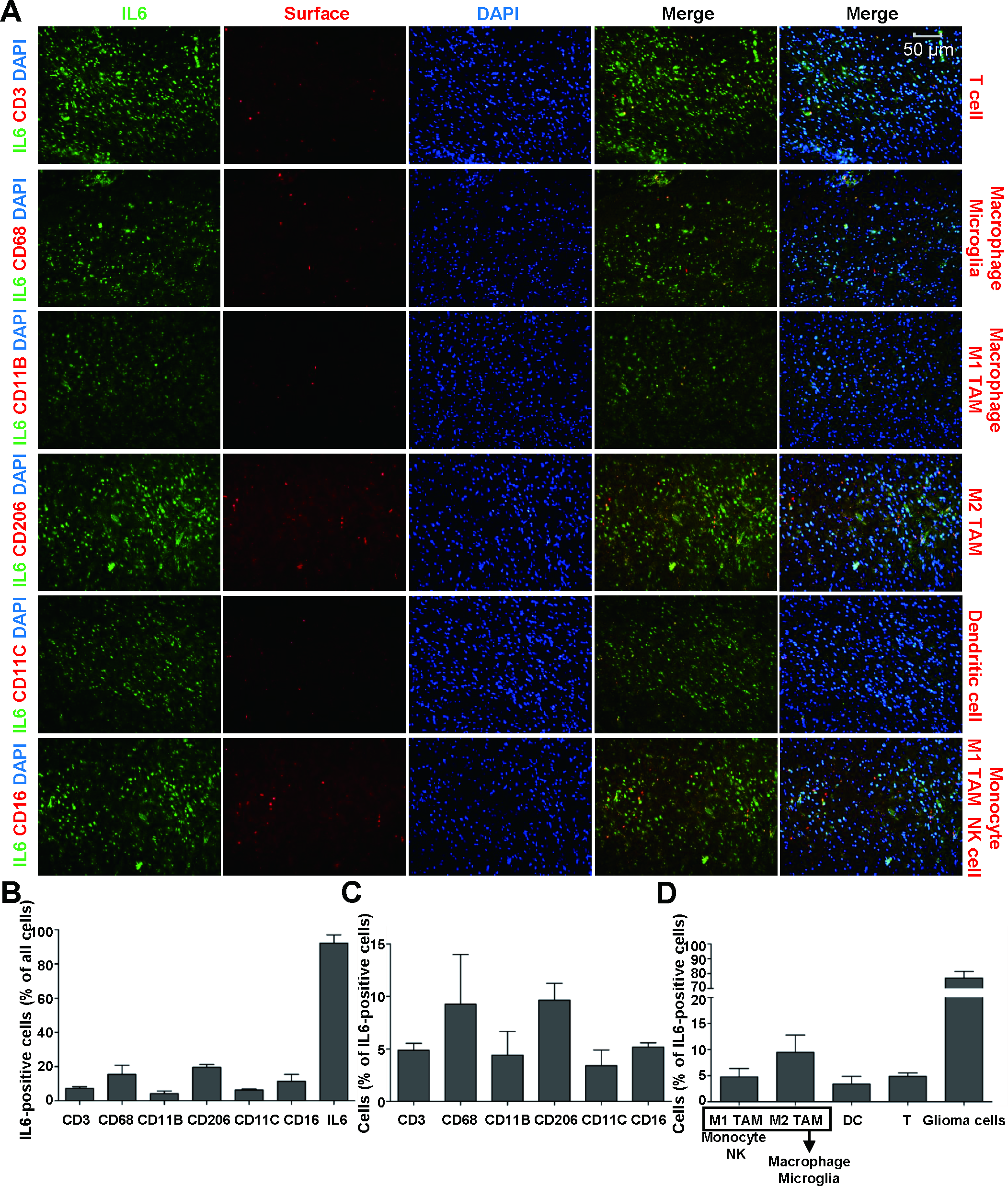

Supplement: KAUP_A_1178446_Supplementary_material.zip [file kaup-12-07-1178446-s001.zip › 2015AUTO0429R2-s03.tif]

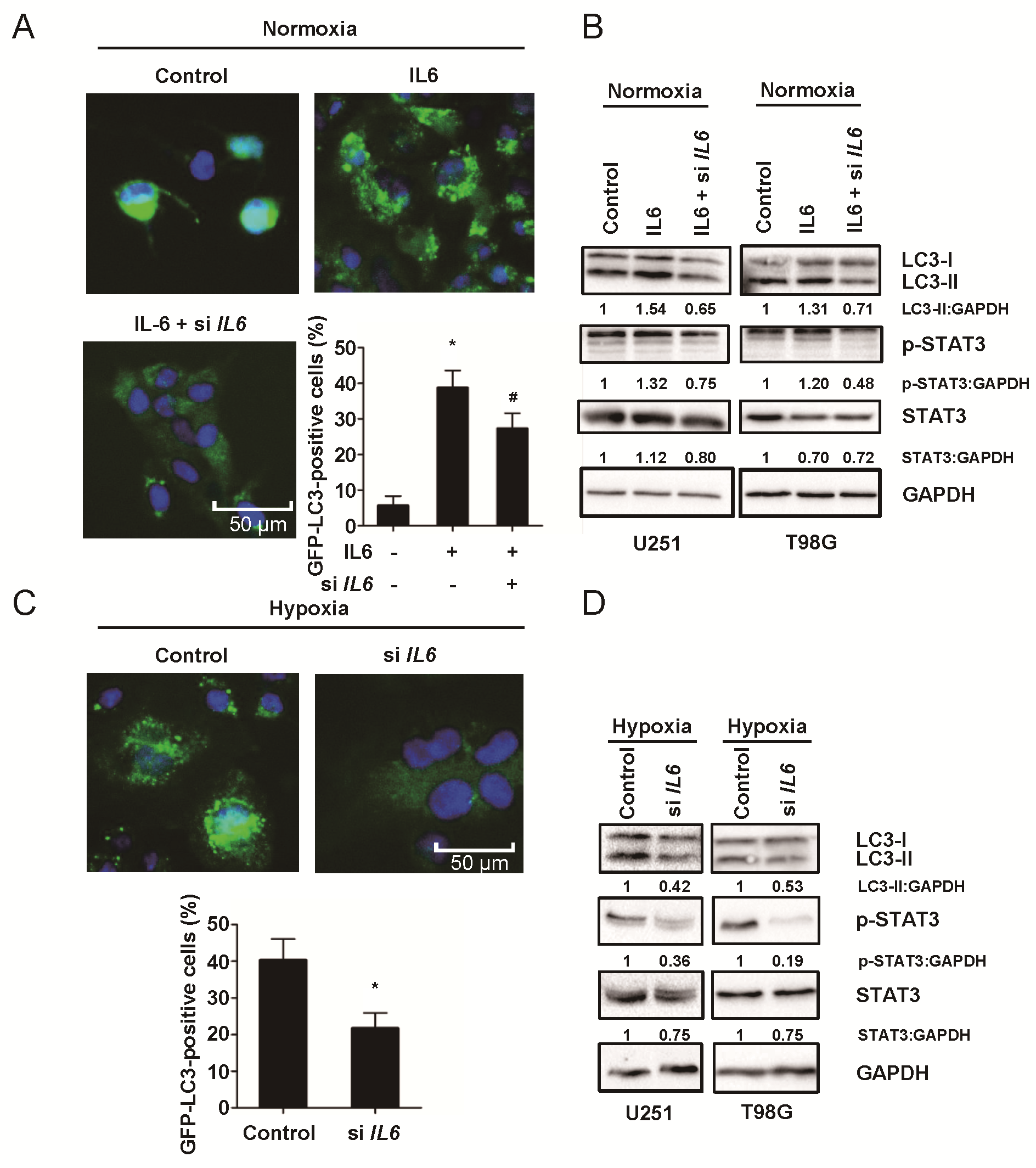

Supplement: KAUP_A_1178446_Supplementary_material.zip [file kaup-12-07-1178446-s001.zip › 2015AUTO0429R2-s04.tif]

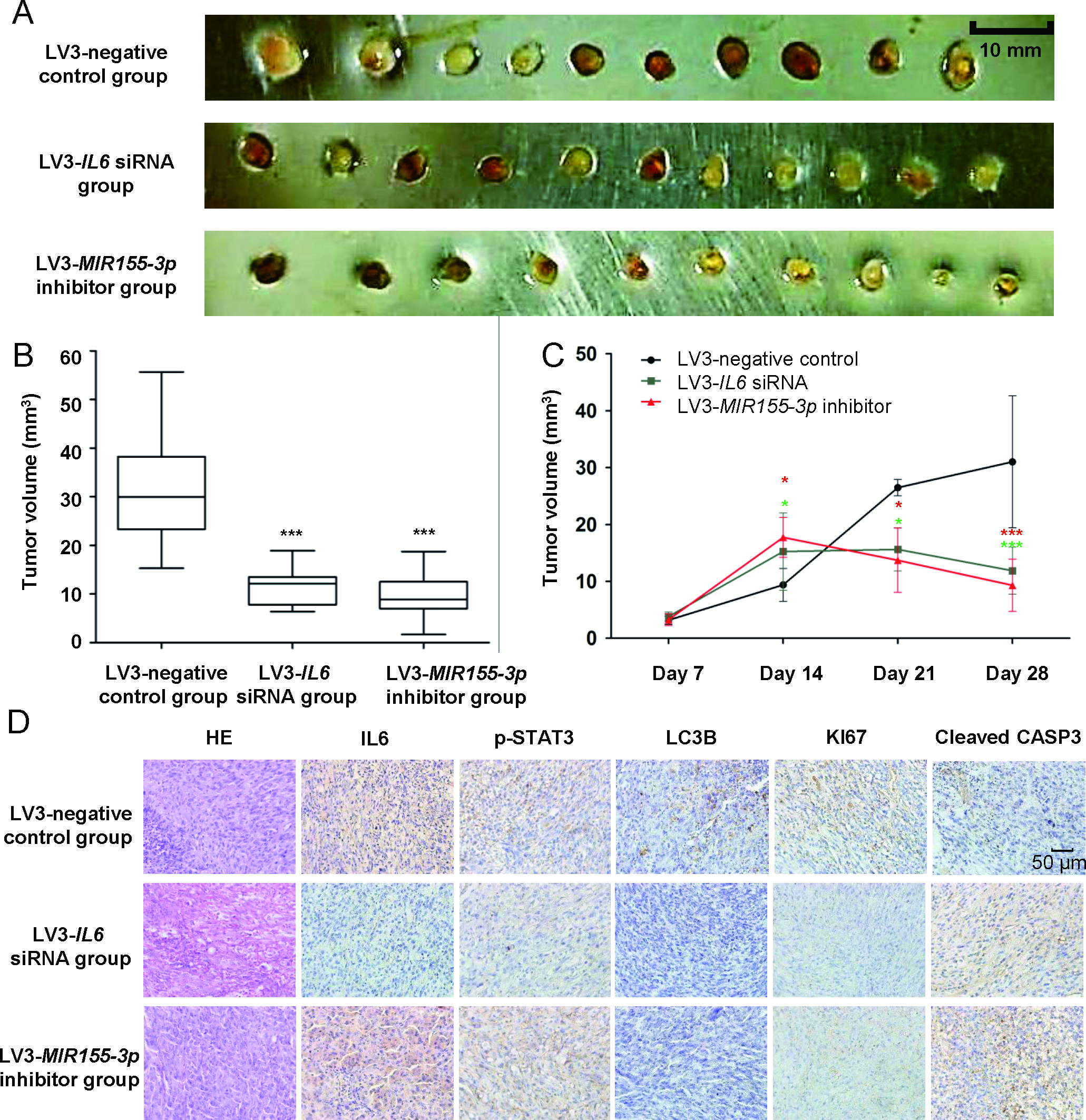

Supplement: KAUP_A_1178446_Supplementary_material.zip [file kaup-12-07-1178446-s001.zip › 2015AUTO0429R2-s05.tif]
